# Effect factors for marine invasion impacts on biodiversity

**DOI:** 10.1007/s11367-024-02325-7

**Published:** 2024-05-30

**Authors:** Philip Gjedde, Fabio Carrer, Johan Berg Pettersen, Francesca Verones

**Affiliations:** Norwegian University of Science and Technology, Trondheim, Norway

**Keywords:** Effect factors, Aliens, MarINvaders

## Abstract

**Purpose:**

Marine vertebrate populations have halved in the past decades, and invasive species are a major driver for this loss. While many model the spread of invasive species, a model to assess impacts of marine invasions, after introduction, has hitherto been missing. We present the first regionalized effect factors for marine invasions. These factors gauge differences in biodiversity impacts after invasions, enabling life cycle impact assessments to highlight biodiversity impacts from invasive species.

**Methods:**

Alien species are species that are introduced by humans to ecosystems where they are not native. We combine data from the IUCN red list and the MarINvaders database to identify the potentially disappeared fraction of native species within each marine coastal ecoregion after alien introduction. The effect factors indicate the biodiversity impact from invasions per alien introduction. However, the IUCN red list has a performance bias between taxonomic groups, and both the IUCN and the harmonized citizen science data from MarINvaders have a geographic observer’s bias. We address some of this bias by evaluating the number of threatened species per number of assessed species, as well as including machine-learning derived data for data deficient species.

**Results and discussion:**

The resulting regional effect factors demonstrate high effects of invasions at high latitudes, which is in line with other findings. Our approach is founded on continuously growing citizen science data and so reflects the biases and uncertainties that follow with this uneven way of data sampling. On the other hand, the continuous data collection by citizen scientists will improve data coverage and thus improve the model. Vice versa, the model itself may be motivation for citizens scientists to collect more data.

**Conclusion:**

The effect of marine invasions presented herein reflects current global information on the issue viewed in a perspective relevant for life cycle impact assessments. The developed effect factors can be used for further assessments that will aid decision-making for policies, industries, and consumers to work towards minimizing impacts of marine invasions and are developed to be compatible with different relevant fate factors.

**Supplementary Information:**

The online version contains supplementary material available at 10.1007/s11367-024-02325-7.

## Introduction

In the past decades, anthropogenic-induced global biodiversity loss has been more rapid than ever before. This is especially pronounced in the marine biosphere, where marine vertebrate populations declined by 49% between 1970 and 2012 (O’Hara et al. 2019; Zoological Society of London., 2015). This has major impacts on human welfare as three billion people rely on the services of the ocean for their livelihoods, e.g., from fishing and tourism (UN 2022).

Woods et al. (2016) highlight seven major drivers for marine biodiversity loss: climate change, ocean acidification, eutrophication, seabed damage, overexploitation, plastic debris, and invasive species. We focus on the latter, which is recognized as a primary driver of rapid biodiversity change in recent decades, but is completely lacking in life cycle impact assessment (LCIA) (Millennium Ecosystem Assessment 2005; Pyšek et al. 2020).

### Aliens, invasives, and their impacts

Species introduced to new ecosystems through anthropogenic pathways are called *alien* species (Robinson et al. 2016). Once alien species establish themselves, they may have serious impacts on the surrounding ecosystem, for example, through competition, predation, parasitism, or ecosystem engineering (Rilov and Crooks 2009; Blackburn et al. 2014). What is more is that there might be secondary impacts through trophic cascades (Thomsen et al. 2014). Some may also have desirable effects, but many have negative effects on their surroundings, and these species are referred to as *invasive* species, or simply “invasives” (Russell and Blackburn 2017).

### Supporting the right choice

We need tools to assess the environmental impacts of different strategic choices to aid us in taking the most sustainable decisions (US EPA 2011). This study aims to advance the LCIA framework, by establishing effect factors (EFs) for a novel impact category for marine species invasions. One of the most relevant pathways is the unintentional introduction of alien species via shipping (Makowski and Finkl 2019). As such, it is an effect that is part of most transport processes throughout the life cycle of products.

Global assessments in the ecological literature tend to focus on the spread and introductions of aliens and mostly rely on literature regarding impacts of individual invasives, such as reported in Davis (2009). Methods that help stakeholders and policymakers assess invasion *impacts* and establish where impacts are smaller or can be minimized, rather than focusing on spread only, are still needed. Due to the prevalence of transport, the inclusion of marine invasion impacts is crucial in life cycle assessment (LCA). In combination with future fate factors (FFs), the EFs will be able to reveal potential “hotspots” of invasion impacts in product life cycles and indicate where more detailed assessments for potential invasions are needed.

### The gap in existing methods

There are only two known methods regarding invasive species intended for LCIA. The first is a case study focusing on freshwater species, modelling the potentially disappeared fraction (PDF) of native freshwater species per goods shipped across the Rhine–Main–Danube canal (Hanafiah et al. 2013). Due to limited data on the “effect per invasive species introduction” relationship, upscaling from the case study to a global level is not possible (Woods et al. 2016). The second method suggests global and regionalized impacts of invasives in the *terrestrial* environment only (Borgelt et al. 2024). However, marine invasions are of a different nature than those in terrestrial and freshwater habitats because marine habitats are better connected and have higher proportions of generalist consumers than other habitat types and are thus better buffered against species extinctions in general (Anton Andrea et al. 2019).

In line with the two previously mentioned LCIA models, we evaluate biodiversity loss, or the effect, as PDF of native species per introduced alien species. This “effect per introduced alien” can be combined with future fate factors for the introduction of invasive species or one of the numerous assessments of marine alien spread that currently lack a way to assess the relative impact that follows when aliens are introduced in one location compared to another (Keller et al. 2011; Seebens et al. 2013; Sardain et al. 2019; Saebi et al. 2020). By looking at the species endangered by invasives (according to the International Union for Conservation of Nature (IUCN)), we have more data than if we assessed the invasives separately. We can then include the impact variations invasions have in different locations. To our knowledge, this is the first assessment that allows for a global perspective on invasion-induced biodiversity loss relative to other locations.

## Materials and methods

### Regionalized invasion impact from alien introductions

A spatially resolved model is needed to evaluate the vast difference in invasion impacts across our ocean’s ecosystems. Distinguishing between *invasives* and *aliens* is complex in spatial data; species may be invasives in one location but not another, depending on where they cause harm. Therefore, we calculate PDF per *alien* introduction and then evaluate how much of that effect is attributable to *invasives* in this location compared to other locations. Thereby, the PDF of native species per alien introduction implicitly covers those aliens that are also invasives. Assessments focusing on alien introductions can therefore be directly used to assess the impact of invasion compared to other locations without the complication of evaluating if each individual alien has invasive effects.

### Data

Data used for calculating the regionalized invasion effect per alien introduction is taken from the MarINvaders toolkit and IUCN red list of threatened species (Lonka et al. 2021; IUCN 2022; Verones et al. 2023). MarINvaders harmonizes several global marine species databases, showing where each registered species is alien and non-alien and which species are threatened by invasive species according to the IUCN. Species’ distributions are geographically represented in the “marine ecoregions of the world by Spalding et al. (2007), hereupon “ecoregions,” based on species occurrence points (see Lonka et al. 2021). We assume that if a species’ occurrence point is within an ecoregion, the species is present throughout that ecoregion following Spalding’s description of ecoregions as: “Areas of relatively homogeneous species composition, clearly distinct from adjacent systems.” We are aware that this is a simplification, but believe this to be in line with assumptions made for LCIAs in terrestrial ecoregions.

A species’ occurrence is not always recorded as a coordinate and can cover multiple ecoregions, so MarINvaders distinguishes alien distributions as “sighted” and “total,” the first being aliens with coordinate records and the latter without, e.g., recorded as present in “China” or “Gulf of Mexico.” Results are calculated using both “sighted” and “total” aliens, for main results and evaluating uncertainty, respectively.

The IUCN Red List of Threatened species is considered the most comprehensive information source for the conservation status of animal, fungi, and plant species and used as an internationally agreed indicator for the status of global biodiversity (IUCN 2023). Assessed species are systematically classified into nine threat levels based on “a probabilistic assessment of the likelihood that a species in a particular threat category will go extinct within some stated time frame”: not evaluated, data deficient (DD), least concern (LC), near threatened (NT), vulnerable (VU), endangered (EN), critically endangered (CR), extinct in the wild (EW), and extinct (EX) (Mace et al. 2008). The systematic classification is based on a standardized approach giving consistency across individual assessments, thereby enabling comparison across taxa and geography in relation to the threat levels.

The IUCN has assessed the existing threats of 19,081 marine species (IUCN, 2023). The threats causing the threat *levels* are categorized into 12 threat *categories*, with 130 threat subcategories in total, among them also “invasive non–native/alien species/diseases” (IUCN 2013). But invasion often happens simultaneously with other threats and can act synergistically to cause declines or extinctions (Gurevitch and Padilla 2004). Therefore, calculating the effect of invasives as “all threatened species per alien introductions” will overestimate the effect of invasives, because species will simultaneously be threatened by other threats, such as fishing, industrial aquaculture, habitat shifting, or tourism. Therefore, we introduce a weighing parameter that we call “relative threat-frequency” to express that not all of an ecoregion’s total threat level is caused by invasive species if multiple threats are at play.

We considered species that belong to the IUCN threat level categories NT, VU, EN, CR, EW, and EX as threatened species. The reason why we chose to include extinct species is because the model is retrospective. In addition, we included species in DD with information from Borgelt et al. (2022) (see Supplementary Information Sect. 1.2). IUCN data was downloaded in March 2023 with a search query including all threat categories and the five marine habitats: Neritic, Oceanic, Deep Benthic, Intertidal, and Coastal/Supratidal. Their location is defined by merging the data with MarINvaders ecoregion data on the species’ scientific name.

### Model overview

The *effect factor (EF)* in each ecoregion* r* is calculated as the *fraction of potentially disappeared native species (PDF)* in the ecoregion* r* per *number of alien species that have been introduced (N*_*Alien*_*).* The *PDF* is calculated as the *number of native species threatened by invasives* in ecoregion *r* (*N*_*Threatened*_) and divided by the total number of species IUCN has assessed in this ecoregion (*N*_*Assessed*_)*.* The numbers of Alien and Assessed species are taken from MarINvaders. Given that each ecoregion is populated by a different number of species, and each of them is exposed to a different number of threats, a set of weights (the relative weight frequency) *Φ*_*inv*_ is introduced to approximate how much harm invasions cause in an ecoregion compared to how much harm is caused by invasions in other ecoregions.

1 $$E{F}_{r}=\frac{PD{F}_{r}}{{N}_{Alie{n}_{r}}}=\frac{{{N}_{Threatened}}_{r}\cdot {{\Phi }_{inv}}_{r}}{{N}_{Assesse{d}_{r}}}\cdot \frac{1}{{N}_{Alie{n}_{r}}}$$

Very little of the ocean’s biodiversity is assessed, and therefore we cannot know the real number of threatened species in any ecoregion (Hughes et al. 2021). So, we estimate the effect *EF*_*r*_ as the number of threatened species per the number of those that have been assessed. *N*_*Threatened*_ counts species threatened by invasives who are not classified as “least concern” by the IUCN, plus a fraction of the data deficient species *T*_*DD*_ (equation S7). *T*_*DD*_ is the sum of the probability of each data deficient species to be threatened according to Borgelt et al. (2022). For testing, we also calculated alternative EFs by excluding data deficient species in *N*_*Threatened*_.

The weights Φ_inv_ represent the frequency of the invasive species threat in each ecoregion, relative to the total number of threats in the ecoregion.

### Relative threat frequency

Threatened species are often threatened by several threats. We approximate the effect of invasive species within an ecoregion by estimating the frequency of native species interacting with invasive species.

Specifically, Φ_inv_ in an ecoregion can be calculated with these steps:

- Calculating the threat intensity of invasive species: The area of a marine ecoregion divided by the sum of the habitat ranges of all native species that are (i) extant within and (ii) threatened by invasive species.
  - Exclude the part of any marine ecoregion that is land.
  - The area of each species’ potential habitat range is calculated as the sum of the areas of marine ecoregions where an occurrence of that species exists. This assumes that invasive species are uniformly distributed within the ecoregion area.
- Analogously, calculate the threat intensity for all the other threats included in the IUCN assessments, e.g., pollution, climate change, and fishing.
- In each ecoregion, the threat intensities are normalized to each other giving a relative value of each threat intensity from 0 to 1 that indicates how much of the total threat in the ecoregion is from each threat.
- Φ_inv_ in an ecoregion is the normalized threat intensity of the threat “invasive non–native/alien species/diseases” specifically.

The complete mathematical derivation of all Φ_inv_ by matrix calculations is described in the Supplementary Information (Sect. 1.1).

## Results

### Regionalized effect

**Fig. 1:**
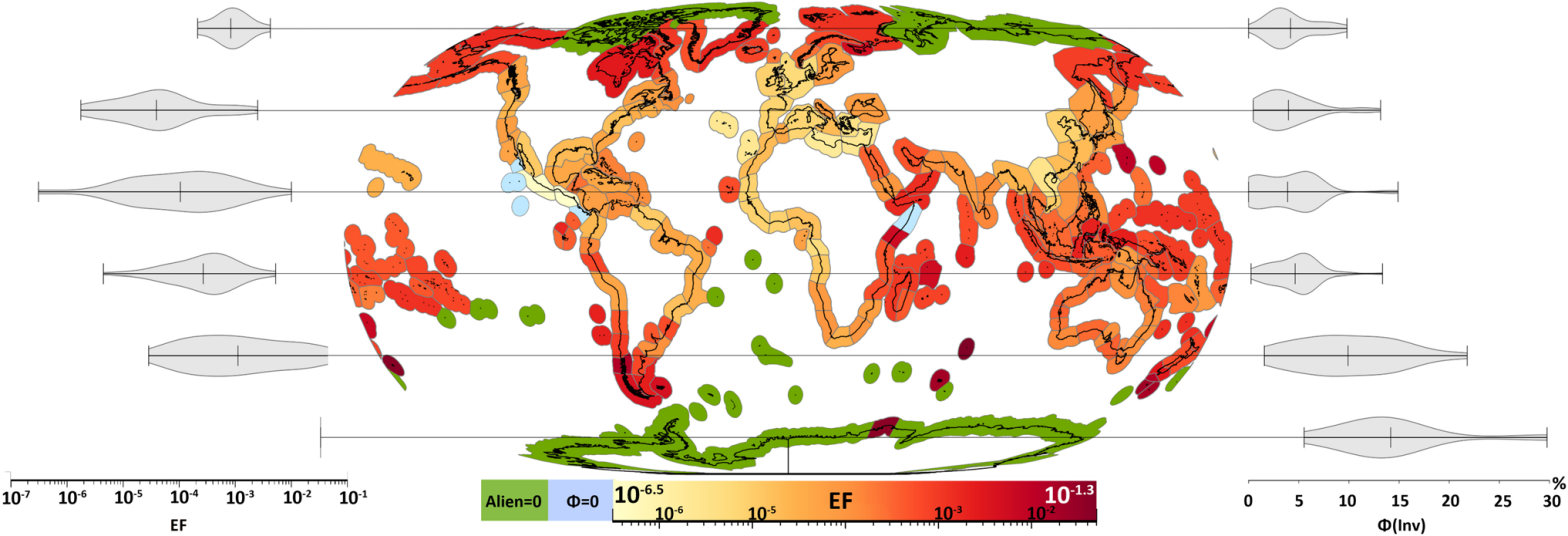
The effect on marine ecosystems per alien species introduction. Blue ecoregions have EF = 0 because there is no threat specifically from invasion in the ecoregion, and green ecoregions are zero because there are no records of alien occurrences. Violin plots on the sides show the distribution of EF (left) and Φ_inv_ (right) for all longitudes within intervals of 30 latitudinal degrees from 90 (north) to -90 (south)

The regional EFs (Fig. 1) range from 0 to 0.05 PDF.Alien^–1^. Such regional variation demonstrates the importance of regionalized assessments. Some ecoregions have EFs of 0 because *Φ*_*inv*_ or number of sighted aliens is 0, meaning that there is no invasive effect from aliens in the ecoregion known yet. *Φ*_*inv*_ of 0 means that there is no species threatened by invasives, so even if aliens are present, there are no species currently known to be threatened by invasive species in these regions, according to IUCN’s classifications. The relative frequency of the invasion threat in one region compared to the invasion threat in other ecoregions (*Φ*_*inv*_) stretches from 0 to 30%. Ecoregions of similar frequency also tend to cluster geographically (see Fig. S3). The pattern of *Φ*_*inv*_ is generally passed on to the regionalized EFs where* Φ*_*inv*_ is extreme and explains low values at the west coasts of Mexico and east coast of Asia.

The expected mean effect of marine invasions is higher in high-latitude regions compared to equatorial regions and lowest in the northern temperate zone (see Fig. 1). The high effect in the Southern Ocean can likely be credited to a high Φ_inv_. Ecoregions in the north also have high EFs relative to more central latitudes, but here, Φ_inv_ is much smaller than in the south, indicating that although there are less species threatened specifically by invasives, there is still many threatened species per introduced alien. The two alternative EFs (i) using “total” aliens from MarINvaders and (ii) disregarding data deficient species can be found along with all results in Supplementary Information (Table S1, and Fig. S4) and show lower impacts per alien introduction in every ecoregion feasible for calculation. The latter of the mentioned alternatives has nearly as high EFs, but EFs using “total” aliens from MarINvaders are on average 0.0014 lower with the biggest gap being 0.05 in the ecoregion “Amsterdam-St Paul” (Fig. S4 and Table S1).

## Discussion

### Model bias

MarINvaders is based on citizen science data and geographically biased because easily accessible locations are better represented (Millar et al. 2019; Hughes et al. 2021). We expect densely populated and research–intensive locations to be given more attention than pristine and remote locations. This is especially relevant at high latitudes (9–198 assessed species compared to a global median of 496). An ecoregion’s initial IUCN assessments may be prioritized based on a notion that the species under investigation is threatened, i.e., observer bias. Therefore, regions with fewer assessments will have higher rates of threatened species per assessed species compared to if all assessed species were randomly selected (see also Table S1). Negative relationships between numbers of assessed and threatened species have also been found by Christie et al. (2021). However, this phenomenon leads to a more cautious approach in less studied, often pristine, ecoregions—and to decrease a region’s EF, a possible approach is to invest in more research leading to either more certainty of the already high EF or a lower EF. Indonesia is an example of a realm with many assessments, but has EFs mostly above the median, indicating that more assessments do not necessarily decrease EFs, even though we may expect higher EFs in Indonesia due to their many endemic species and the correlation between endemism and ecosystem vulnerability (Berglund et al. 2009).

Apart from geographic biases, MarINvaders and IUCN also face a taxonomic bias, since they include mostly species from higher taxa that are well-studied, but we partly mitigate this by calculating each EF relatively as *per assessed species* (Lepczyk et al. 2022). We also include machine learning data from Borgelt et al. (2022) for broader taxonomic and geographic coverage (see Supplementary Information (Sect. 1.2)).

### Sensitivity and data uncertainty of alien and threatened species counts

Equation 1 consists of four variables: *N*_*Threatened*_, *Φ*_*inv*_, *N*_*Assessed*_, and *N*_*Alien*_. The first two affect the output linearly, but the latter two cause greater output change per input at low values, meaning that we expect more extreme EFs from pristine or unresearched regions. In addition, number of aliens (*N*_*Alien*_) and number of species estimated threatened (*N*_*Threatened*_) have higher data uncertainty. With the difference of “total” and “sighted” aliens presented by MarINvaders follows the uncertainty that some alien species may have been sighted outside the “sighted” ecoregions, but not recorded with coordinates. In conjunction with the model being sensitive at low values of *N*_*Alien*_, it is not surprising that we see significantly lower EFs when including “total” aliens. This also indicates the model’s sensitivity to the precision of species’ habitat ranges, highlighting how accuracy can be improved by improving this parameter both in Eq. (1) and in the derivation of *Φ*_*inv*_ (Supplementary Information Sect. 1.1).

Following Eq. (1), green ecoregions in Fig. 1 are n/a values due to division of zero aliens, i.e., almost no aliens are reported in the Southern Ocean. But all Southern Ocean ecoregions have a high *Φ*_*inv*_, indicating that the first alien occurrence point immediately leads to a high EF. Since both the Arctic and Antarctic regions are more prone to invasions due to global climate change and increasing human traffic, our findings suggests that invasions in high latitudes are concerning for future scenarios, a finding that is supported by other studies (Bennett et al. 2015; Hughes et al. 2020). EFs in high latitudes are also high due to many species being threatened by few aliens which indicates high invasion potential by the aliens or high invasion vulnerability in ecosystems of these latitudes. Some assessments, such as those used for conservation plans for marine protected areas, are not streamlined, and do not always include invasive species, e.g., in Canada and Antarctica invasives are often neglected (O’Regan et al. 2021). We now see that assessments of especially Canada and Antarctica would benefit from including invasive species in assessments.

### An example of the sensitivity of regional threat intensity and Φ_inv_

The regional threat intensity (*r,t* element in *I*_*RT*_ in Supplementary Information equation S3) used for *Φ*_*inv*_ is sensitive to the number of threats and habitat ranges of the threatened species, when there are few threatened species in the region. An example is the development in the ecoregion “The Sao Pedro and Sao Paulo Islands” (see Fig. S4) from September 2022 to March 2023. In September 2022, it had a *Φ*_*inv*_ of 22% and 1 known threatened species. *Enneanectes smithi*, a ray-finned fish species, is only extant in this ecoregion and is classified by IUCN as “vulnerable” (VU) under criteria D2, meaning that it has restricted species habitat range and is vulnerable to invasives (IUCN Standards and Petitions Committee 2022).* E. smithi* is assessed as threatened by four threats, one being the infamous invasive red lionfish (*Pterois volitans)*, indicating that the ecoregion’s species indeed was vulnerable to invasives thus far. In March 2023, the ecoregion had *Φ*_*inv*_ of 14% and 3 threatened species, *E. smithi* still among them, while the other two are coral species (*Madracis decactis* and *Scolymia wellsii*) with more threats and larger habitat range. The EF of the region went from 0.004 to 0.0015—a decrease of EF despite an increase of threatened species occurrences. This is explained by the decrease of *Φ*_*inv*_ and effect of the new threatened species having more diverse threats.

### Limitations using global IUCN threat assessments

When *Φ*_*inv*_ evaluates the threat of invasion, it does not differentiate if the threat is from a marine or terrestrial invasive, e.g., some of the threat may be from cats preying on nests of marine birds. Another limitation is only using global assessments instead of regional assessments. Global assessments may classify a species as threatened even when it is not threatened in all regions. On the other hand, global assessments may not label regionally threatened species as threatened on a global scale. Having regionalized assessments would therefore increase accuracy, but such data is lacking on a global scale. Future studies should therefore evaluate the local threat to extinction for comparison.

Some IUCN assessments detail the magnitude of each threat as an “impact score” (ranging 0–9), but only 51% of species–threat code combinations have such a score. Due to inconsistencies of this detail in the data, we have not included this for the modelling of *Φ*_*inv*_.

### Limitations to practical use based on current shortcomings

We have discussed observer, and taxonomic biases from the IUCN, and model variables that are sensitive to low values of *N*_assessed_ and *N*_Alien_. Simultaneously, the mentioned biases affect the sensitive variables especially in pristine regions. In addition, using global IUCN assessments is not optimal for assessing the impact on a regional scale, and the Φ_inv_ is currently but a rough estimate of scaling the total threat to biodiversity down to that caused only by invasive species. Lastly, quantifying uncertainty is infeasible, as IUCN assessments do not have quantified uncertainties. Like most new models shy on reliable data, these shortcomings should be considered when using this model. These EFs are at best a conservative estimate, but they still present regions at high risk relative to other regions. This enables us to point out exchanges in value chains that require more attention in relation to invasive species and that makes these EFs useful in LCA because LCA assesses our trade network through value chains well.

### Perspective to previous assessment of invasive species

The results in Fig. 1 contrast, to our knowledge, with the only previous global assessment of marine invasive species. Molnar et al. (2008) mapped the distribution of 329 marine invasive species and assessed the global threat of invasions based on a threat–scoring system evaluating each invasive species. This is useful for conservation by protecting against certain species, but it does not include impacts from unknown invasives, and any further development of the assessment requires individual assessment of each invasive species in each assessed region. In addition, the perspective of Molnar et al. is that high levels of invasion are caused by *high numbers of invasive species with high threat scores*. In contrast, we evaluate the relative vulnerability or invasibility between ecoregions based on *how much threat to native species* has been recorded. So, where Molnar et al. presents the current state of recorded biological invasions, we present the recorded state of damage (effect) biological invasions have caused up to this point in time.

The difference between Molnar’s and our results indicates either both or one of the following statements: (i) There is a research bias in both our and Molnar’s results, as discussed above. (ii) Neither a higher number of invasives nor their perceived individual “dangerousness” alone, as evaluated in Molnar et al. (2008), leads to a higher number of species threatened by invasives. The latter statement is also supported in our results by regions, such as “Amsterdam–St Paul” and “Chatham Island” (both Southern hemisphere island groups (see Fig. S3), each with only one alien sightings: *Hypnea musciformis* and *Mytilus galloprovincialis*, respectively, and yet high EFs. Our results show that the ecoregions are vulnerable to invasives by having ~ 11 and ~ 24 threatened species (including data deficient species) despite only one recorded alien sighting. While threatened species may meet their threat in other ecoregions, the general trend of high *Φ*_*inv*_ in the Southern Ocean rather indicates that it is either a lack of data on present aliens *or* that the one alien present has considerable, detrimental invasion effects.

### Fate modeling: an outlook

Aliens can be intentionally or unintentionally introduced. Unintentional introductions in marine ecosystems mainly happen through ballast water in ships or due to hull fouling. Ballast water provides stability and helps with maneuvering and is pumped in and out of the ship’s ballast tanks adjusting to the cargo on board. Ninety percent of all world trade is based on ship transport, making this a crucial impact pathway (Molnar et al. 2008; Makowski and Finkl 2019; Pyšek et al. 2020; IMO 2022). Species that can transport themselves on the hull of ships may additionally also “hitchhike” on marine debris, raising concerns that they reach pristine environments without our knowledge (García-Gómez et al*.*, 2021). Future work should incorporate these pathways into the LCA framework.

Other relevant pathways worthy of mentioning are aliens that are intentionally introduced, released, or escaped from aquaria, or introduced with seafood trade. In addition, the same alien species may have spread using multiple different pathways, both intentional and unintentional, as is speculated for the infamous European Green Crab, *Carcinus maenas* (Cohen et al. 1995). Unintentional introduction of invasives are heavily driven by propagule pressure, that is, the constant introduction of alien propagules over time increases the probability of a successful introduction (Davis 2009). But introductions that are intentional, from release, or escape are often of mature individuals and therefore rely less on propagule pressure and can also not be modelled as a probability like unintentional introductions or “pressure” pathways like shipping.

It is difficult, if not impossible, to accurately map the pathways causing invasive spread of one species. However, a statistical estimate of introductions from the most relevant pathways, in terms of quantities of species, is both feasible and useful for LCA when linked with the EFs. That is, future fate factors must model the introductions of invasive species through different pathways per elementary flows in life cycle inventories. And then, such FFs combined with these EFs will enable us to assess and compare multiple different pathways, and different choices, in LCA. As such, these EFs can be a baseline for invasion impacts on marine ecosystems when comparing different pathways (FFs) in different regions.

## Conclusion

Better tools are needed for decision-makers to mitigate marine invasions. Quantifying the impact of invasive species by invasiveness of the species is not as descriptive of the recipient ecosystem as the invasibility of that ecosystem. We focus for the first time on that perspective, on a global, yet regionalized scale. We find that invasion effects, the number of threatened species per introduced alien, is greater in high latitudes and on islands meaning that these ecoregions should be of a higher priority in avoiding invasions compared to other ecoregions. Simultaneously, these ecoregions are poorly represented in number of assessed species which along with research biases may exaggerate the EFs. However, this results in a conservative approach that rather protects and researches pristine areas too much over too little.

The EFs are more useful for decision support when linked to human activities, through future FFs. They can act as “common ground” for multiple fate factors in an impact assessment framework for marine invasions. Human activities known to result in the spread of invasive species are for example international shipping, horticultural or pet trade, recreational watercrafts, or “hitchhikers” on floating marine debris. This method connects to all these pathways and will help us better understand their impacts.

Current data for this model are sparse and biased, but also improving at an exponential rate. But data improve faster when there is a public interest or an incentive for politicians or the industry to improve it. We believe that the introduction of biological invasion impacts on marine biodiversity to the LCA framework further encourages such improvements. Therefore, a model that reflects the data genuinely, without distorting it with subjective weights and bias accounting, also improves with data, and in this case when the model is used.

### Supplementary Information

Below is the link to the electronic supplementary material.

Supplementary file1 (DOCX 1660 KB)

## Data Availability

We used Python 3.10.5, NumPy 1.23.0, Pandas 1.4.3, and MarINvaders 0.4.1 for calculations, while ArcGIS pro 3.0.2 and matplotlib 3.8.2 were used for plotting figures and ArcGIS pro for computing the geodesic area of each ecoregion, using the equal–area projected coordinate system: Equal Earth (sphere). See the GitLab project (https://gitlab.com/marinvaders/marinvaders/-/tree/master/utility) for data acquisition and python code.
