# Supplementary material for: Effect factors for marine invasion impacts on biodiversity
Source: Int J Life Cycle Assess. 2024 May 30;29(9):1756–63. doi: 10.1007/s11367-024-02325-7 (PMC11358188; doi:10.1007/s11367-024-02325-7)
Supplement: Supplementary file 1 — Supplementary file1 (DOCX 1660 KB) [file 11367_2024_2325_MOESM1_ESM.docx]

Supplementary Material

# 1 Method’s extension

## 1.1 Derivation of Φ_inv_

We estimated a weighted frequency of one threat in a region relative to other regions. This relative frequency, specifically for the threat of invasion, is used to approximate how much invasions cause harm in an ecoregion relative to the harm caused by invasions in other ecoregions. We assume that impacts of individual threatening events (such as invasions) would be normally distributed around their mean impact. Following the central limit theorem, the frequency of events can represent the impact of such types of events.

Let *S* be the total number of both native and non-native species, *R* the number of ecoregions. A binary matrix with dimensions species × ecoregions (*B_SR_*) represents in which ecoregion each species is listed as extant. The vector $\overline{\omega}_{S}$ is the total area populated by each species, obtained by multiplying *B_SR_* with a vector consisting of the area of each ecoregion $\overline{\alpha}_{R}$.

$$\begin{aligned} \begin{aligned} \overline{\omega}_{S}=B_{SR}\cdot\overline{\alpha}_{R} \end{aligned}\# (S1) \end{aligned}$$

The subscripts *S* and *R* indicate the length of the vectors  $\bar{\omega}$ and  $\bar{\alpha}$ respectively.

We need to count how many times invasive species are a threat, compared to the total number of threats within an ecoregion. We assume that the threat impacting a species is uniformly distributed within the ecoregions where the species is present. Therefore, we weigh the threat to each species present in the ecoregion on the principle that small species ranges are better indicators for the presence of a threat than large species ranges (Eq. 2). Let *I_RS_* be an ecoregion × species matrix whose elements (*r,s*) are the *r*-th element of $\overline{\alpha_{R}}$, the area of ecoregion *r*, divided by the *s-*th element of $\overline{\omega_{S}}$, the habitat range of species *s*. *S_r_* is species *s* inhabiting ecoregion *r*, so if species *s* is not present in ecoregion *r*, then the corresponding element is set to 0.

$$\begin{aligned} \left( I_{RS} \right)_{r,s}=\left\{ \begin{aligned} \alpha_{r}\cdot\omega_{s}^{-1}, s\in S_{r} \\ 0 , &s\notin S_{r}\text{ } \end{aligned} \right.\#(S2) \end{aligned}$$

It follows that each element (r,s) of the I_RS_ matrix represents the ratio of the ecoregion r and the habitat range of species s, provided that the species populates that ecoregion. This scaling serves the purpose of making ecoregions with different areas comparable.

To find the threat frequency for all regions and threats, let *T* be the number of threats and *B_ST_* a species × threats binary matrix, being the threats indexing corresponding to the IUCN threat code. The matrix product $I_{RS}\cdot B_{ST}$ then results in the regions × threats matrix $I_{RT}$, whose elements represent the weighted frequency of each threat in each ecoregion.

$\begin{aligned} \begin{aligned} I_{RT}= I_{RS}\cdot B_{ST} \end{aligned}\#(S3) \end{aligned}$

Figure S1 exemplifies the *I_RS_* for seven example regions *r* in South America and four example species threatened by invasion. The summed elements of *I_RS_* represent the *threat frequency* presented in *I_RT_*; how often this threat is met by species in *r* assuming the spatially uniform threat distribution.


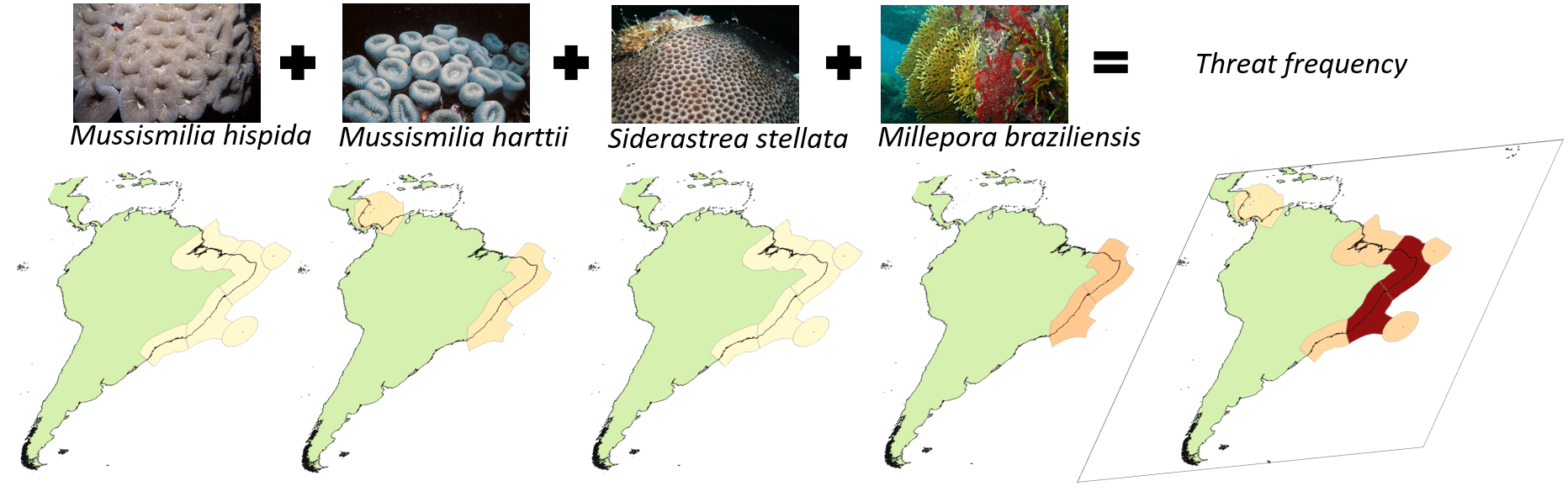


**Figure S1:** Visualisation of weighed threat of four South American species, each being a combination of elements of the I_RS_ matrix. When summed they are the threat frequency, i.e., the sum is one element of the I_RT_. The intensity of the threat frequency is indicated as weak to strong from yellow to red. Pictures are sourced from Veron et al. (2016), except Millepora braziliensis which is pictured as Monanchora braziliensis under license from shutterstock.com.

Lastly, every value in the $I_{RT}$ matrix is normalized with respect to the sum of the other values in the row thereby representing a proxy of how frequent each threat is in each ecoregion compared to the other threats.

$$\begin{aligned} \begin{aligned} \left( RF_{RT} \right)_{r,t}=\frac{\left( I_{RT} \right)_{r,t}}{\sum_{t=1}^{T} \left( I_{RT} \right)_{r,t}}, r\in\left\{ 1,..,R \right\}, t\in\left\{ 1,..,T \right\} \end{aligned}(S4) \end{aligned}$$

Amongst the 130 threat-categories described in the IUCN red list, the impact of each specific threat varies greatly. It therefore becomes important to consider how we normalize the *I_RT_* rows to fairly represent the threat frequencies. The specific threat “invasive non-native/alien species/diseases” has threat-code 8.1 and is amongst six sub-categories (8.1-8.6) for the general threat (8) “Invasive and other problematic species, genes & diseases”. Threat code 8.1 further has sub-sub threat categories 8.1.1 and 8.1.2. If we normalize on 130 threats in *RF_RT_* we assume a similar importance to each threat although the very system of classifying threats into categories and sub-categories indicates different importance. Therefore, instead of normalizing on 130 threat codes, we first normalize on the 12 major IUCN threat codes that include many sub-categories and then on the sub-categories of the major category. As an example, the threat frequency from the major category 8 “Invasive and other problematic species, genes & diseases” in Southern Norway is 11%. Then we normalize again on the 6 sub-threats belonging to threat category 8 and find that threat code 8.1 (Invasive non-native/alien species/diseases) makes up 33% of the major threat code 8. The threat frequency of 8.1 for Southern Norway is therefore 11% of 33%, so 4%. If we initially normalized on all 130 threat codes, by having major and sub-categories in one bunch, the threat frequency in Southern Norway would be 2.4%. We give more credit to the relevance of threat code 8.1 within threat code 8, compared to codes 8.2-8.6. Figure S2 shows a comparison of normalizing on 12 plus 130 threat codes (142) instead of the 12 major- and then the sub-categories. These results show similar patterns (high R^2^), but normalizing on 12 major- and then 6 sub-categories always result in a higher Φ_inv_. The sub-vector of *RF_RT_* representing threat code 8.1 is our proxy of regional invasion vulnerability Φ_inv_.

$$\begin{aligned} \left( \Phi_{inv} \right)_{r}=\left( RF_{RT} \right)_{r,t} , r\in\left\{ 1,.., R \right\}, t\in\left\{ 8.1 \right\}\#(S5) \end{aligned}$$

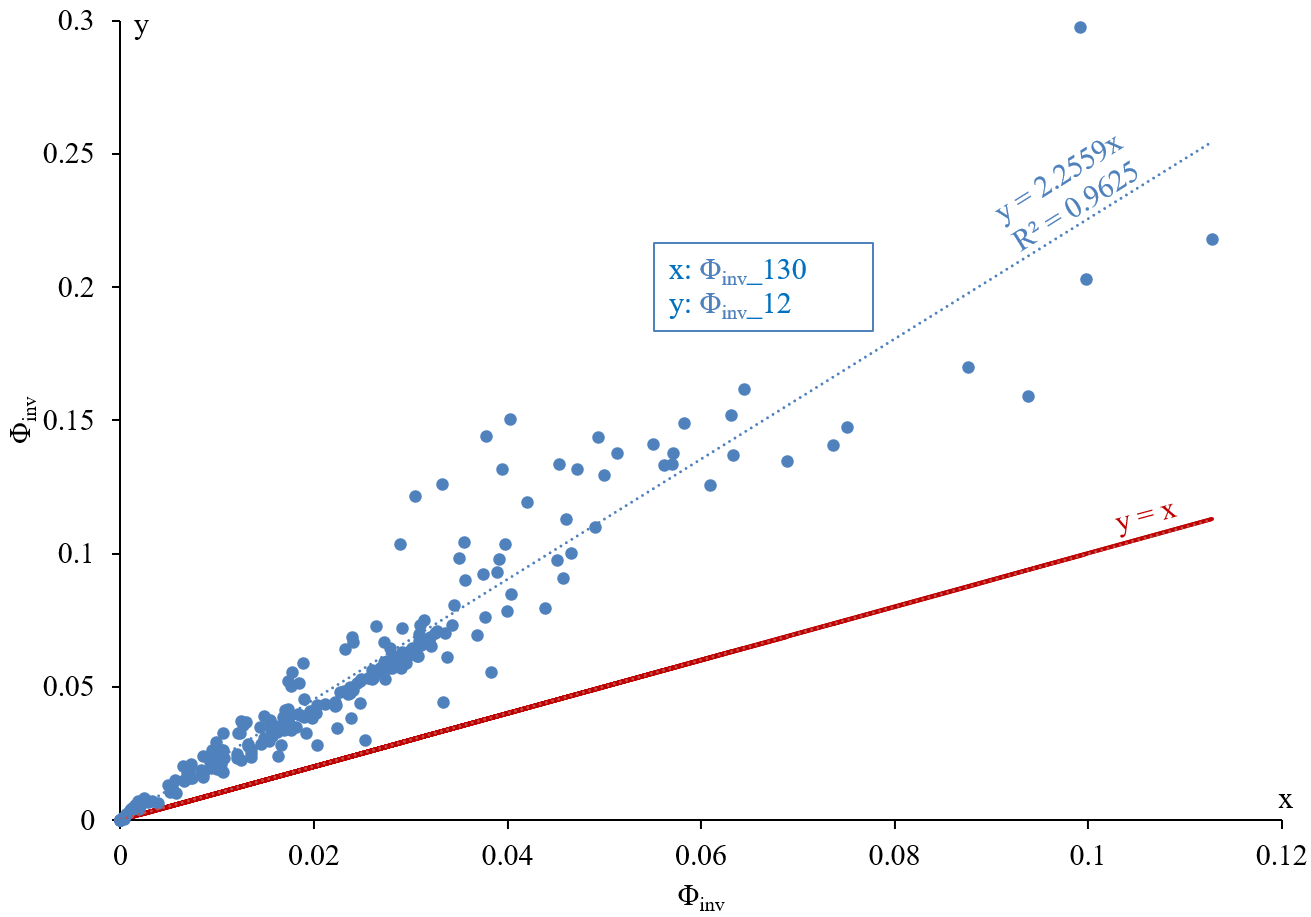
 ***Figure S2:*** *Comparison of Φ_inv_ in all ecoregions depending on whether Φ_inv_ is normalized to 12 major threat-categories and then 6 sub-categories (8.1-8.6), or if the Φ_inv_ has been normalized on 12 major- plus 130 sub-categories in one go. Both the x- and y-axis show Φ_inv_*. But the blue scatter plot shows the relationship between normalizing on 12 major plus 130 categories (x) or 12 and then 6 (y) and that normalizing on 12 and then 6 is conservative as it always gives a higher *Φ_inv_* than the *Φ_inv_* on 12 major- plus 130 sub-categories.

## 1.2 Estimation of threatened species from data deficient assessments

Amongst 19,081 marine species assessed in the IUCN red list 12,797 are “least concern”. 6,284 are somehow impacted (threatened or extinct), where 3,376 are data deficient with unknown extinction-risk. Borgelt et al. (2022) trained a supervised machine learning model to classify threatened species and discovered that half of the data deficient species were likely to be threatened. We divided endangered species in two groups (Eq. 6): 1) *T_IUCN_* are categorized as threatened by belonging to any IUCN red list category except “least concern” or “data deficient”. 2) *T_DD_* are species in the data deficient group who are threatened (Eq. 7). The two groups’ sum is the total estimate of endangered species *N_Threatened_* in region *r*.

$$\begin{aligned} \begin{aligned} N_{Threatened_{r}}=T_{IUCN_{r}}+T_{DD_{r}} \end{aligned}\#(S6) \end{aligned}$$

To find *T_DD,_* we first include the threat probability of each species estimated by Borgelt et al. (2022) and sum up their contributions. Let *P(T_i_)* be the threat probability of species *i* in a group with *Fe* number of species that was *feasible* for assessment by Borgelt et al. (2022). The number *Ie* species were *infeasible* for assessment by Borgelt et al. (2022). So secondly, we add the mean value of *P(T)* of the feasible species multiplied by *Ie*. This fills out the gaps of assessment-infeasible species with an estimate closer to the true value of threatened species than if all or none of the data deficient species had been included. *T_DD_* is then the fraction of data deficient species who we estimate to be threatened in each ecoregion *r*.

$$\begin{aligned} T_{DD_{r}}=\sum_{i=1}^{Fe_{r}} \left( P\left( T_{i} \right) \right)_{r}+\frac{\sum_{i=1}^{Fe_{r}} \left( P\left( T_{i} \right) \right)_{r}}{Fe_{r}}\cdot Ie_{r} \#(S7) \end{aligned}$$

# 2 Other supporting figures


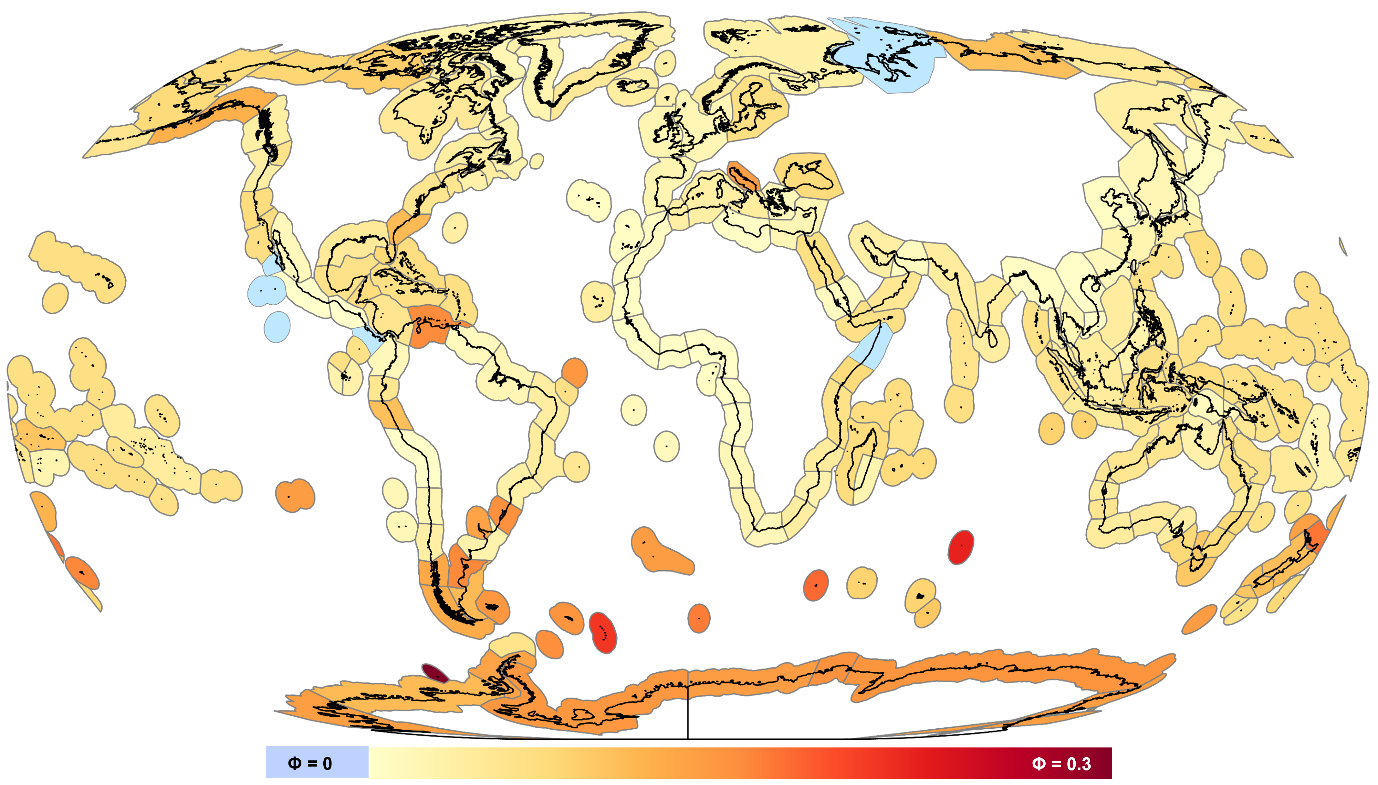


***Figure S3:*** *Φ_inv_ for all ecoregions ranging from 0-0.3. Blue ecoregions have no native species threatened by invasive species. The higher the Φ_inv_ value, the more of the general threat in this ecoregion is represented by invasive species.*

It is important to realise in Figure S3 that we have used global assessments from the IUCN red list. Thereby we have some regions where native species are assessed as threatened by invasive species even though we have seen from the MarINvaders data that there are no records of alien species. This is especially the case in many Southern Ocean ecoregions. In these cases, species within the ecoregion have previously shown traits of invasibility in other regions, indicating that they are easily impacted by invasive species. Therefore, we expect high impacts when the first aliens with invasive traits are introduced to these environments.


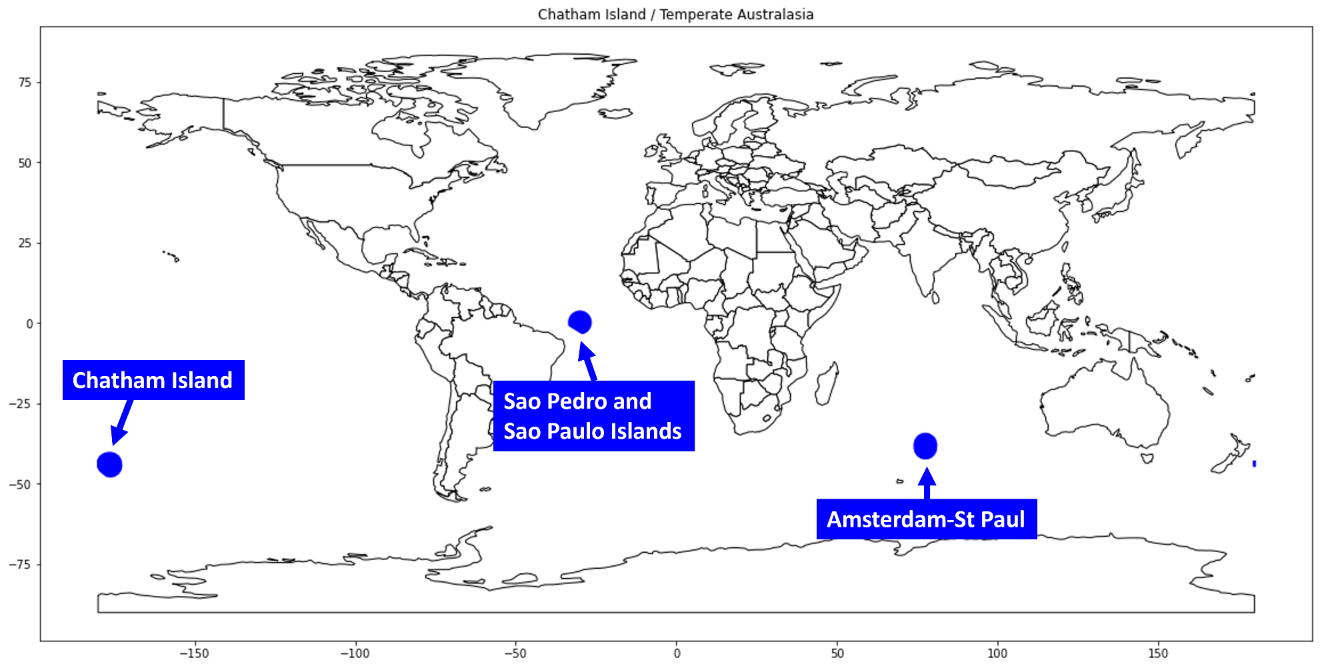


***Figure S4:*** *The locations of islands mentioned in the article.*

The islands “Chatham Island” and “Amsterdam-St Paul” in figure S4 have very high EFs despite inhabiting only 1 alien species. This indicates that one invasive species can indeed have detrimental effects on native ecosystems. The Sao Paulo Islands also used to have higher EFs than what is shown in the final model results. The EF decrease in this ecoregion owes to the fact that there are now records of 3 threatened species compared to the earlier 1, which with no change of invasive species intuitively would increase the EF. But the two new threatened species are threatened by more threats than just invasive species. This reveals that there are more threats to this ecosystem than just invasive species, and so the impact that is specifically from invasive species is now lower because we divide the total impact (3 threatened species) over more different threats, leaving less of the blame left on invasive species.


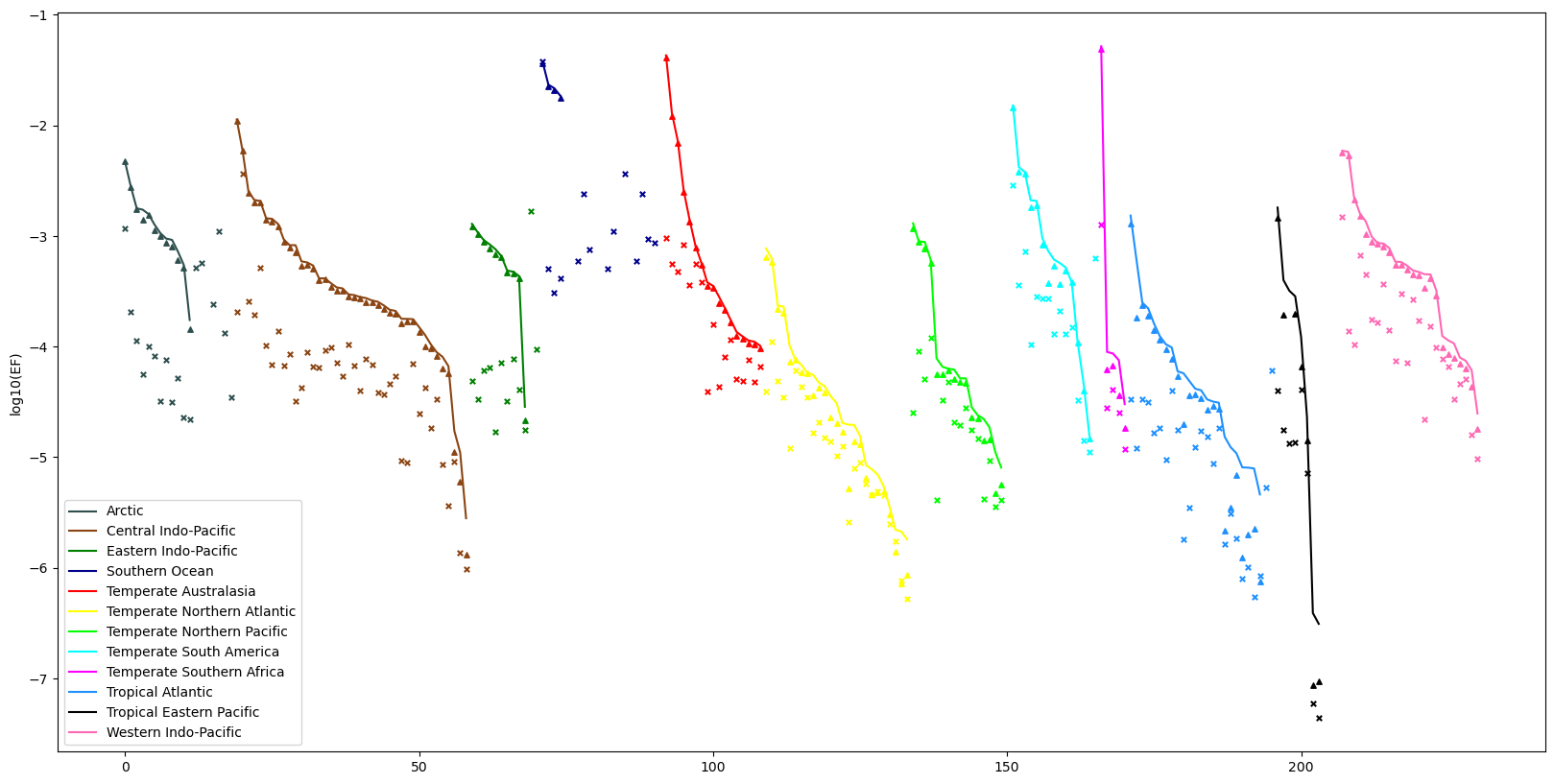


***Figure S4:*** *Main and alternative effect factors in log10(EF). We sorted EFs from largest to smallest within each eco-realm. The main effect factors presented in the article (line-plots) along with the two alternative calculations of the effect factors (i; crosses “x” and ii; triangles “^”). (i) Crosses “x”: Effect factors calculated using alien observations with qualitative description of location (e.g., “South-Pacific Ocean” or “Australia”), and not only observations with given coordinates or ecoregions (as is the case for the main effect factors). (ii) Triangles “^”: Effect factors calculated without the inclusion of any data deficient (DD) species. The main effect factors we do as described in the chapter in the main article on “Estimation of threatened species from data deficient assessments”.*

**Note** that the effect factors are in a negative logarithmic scale, therefore the higher the negative logarithmic number, the lower the effect factor value. *I.e.,* the smallest effect factors are around 7 and the largest around 1. The main effect factors presented in the article (*blue*) is always a higher or similar value to the alternatively calculated effect factors.

# 3 Effect factor table overview

*Table S1: Effect factor table with “normal” and “log10” values of the main EFs and the two alternatives used for discussing uncertainty (“excl. DD”, and “aliens (total)”) and the variables leading to these EF values.*

| **ECO**  **CODE** | **ECOREGION** | **REALM** | **total**  **species** | **aliens** | **alien**  **(sight)** | **threatened**  **(excl. DD)** | **threatened**  **(incl. DD)** | **Φ_inv** | **assessed** | **EF (main)** | **EF (excl.**  **DD)** | **EF aliens**  **(total)** | **‐log10(EF)** | **‐log10(EF)**  **(excl. DD)** | **‐log10(EF)**  **aliens (total)** |
| --- | --- | --- | --- | --- | --- | --- | --- | --- | --- | --- | --- | --- | --- | --- | --- |
| 20192 | Agulhas Bank | Temperate Southern  Africa |  | 43 | 17 | 27 | 27,0 | 0,019 | 823 | 3,6E‐05 | 3,6E‐05 | 1,4E‐05 | 4,4E+00 | 4,4E+00 | 4,8E+00 |
| 20053 | Aleutian Islands | Temperate Northern Pacific | 1106 | 104 | 4 | 13 | 13,3 | 0,051 | 153 | 1,1E‐03 | 1,1E‐03 | 4,3E‐05 | 3,0E+00 | 3,0E+00 | 4,4E+00 |
| 20072 | Amazonia | Tropical Atlantic | 1022 | 110 | 7 | 7 | 8,6 | 0,018 | 373 | 6,0E‐05 | 4,9E‐05 | 3,8E‐06 | 4,2E+00 | 4,3E+00 | 5,4E+00 |
| 20194 | Amsterdam‐St Paul | Temperate Southern Africa | 288 | 41 | 1 | 18 | 19,0 | 0,146 | 144 | 1,9E‐02 | 1,8E‐02 | 4,7E‐04 | 1,7E+00 | 1,7E+00 | 3,3E+00 |
| 20228 | Amundsen/Bellingshause  n Sea | Southern Ocean | 478 |  | 0 |  |  | 0,121 | 25 |  |  |  |  |  |  |
| 20109 | Andaman and Nicobar Islands | Western Indo‐Pacific | 5004 | 25 | 14 | 262 | 263,6 | 0,059 | 1143 | 9,8E‐04 | 9,7E‐04 | 5,5E‐04 | 3,0E+00 | 3,0E+00 | 3,3E+00 |
| 20110 | Andaman Sea Coral Coast | Western Indo‐Pacific | 1789 | 13 | 6 | 112 | 112,0 | 0,034 | 918 | 6,8E‐04 | 6,8E‐04 | 3,2E‐04 | 3,2E+00 | 3,2E+00 | 3,5E+00 |
| 20223 | Antarctic Peninsula | Southern Ocean | 2125 |  | 0 |  |  | 0,131 | 73 |  |  |  |  |  |  |
| 20090 | Arabian (Persian) Gulf | Western Indo‐Pacific | 1006 | 11 | 6 | 49 | 49,1 | 0,021 | 315 | 5,4E‐04 | 5,3E‐04 | 2,9E‐04 | 3,3E+00 | 3,3E+00 | 3,5E+00 |
| 20178 | Araucanian | Temperate South America | 664 | 21 | 5 | 10 | 10,3 | 0,042 | 149 | 5,7E‐04 | 5,6E‐04 | 1,4E‐04 | 3,2E+00 | 3,3E+00 | 3,9E+00 |
| 20140 | Arnhem Coast to Gulf of  Carpenteria | Central Indo‐Pacific | 4736 | 86 | 15 | 125 | 125,0 | 0,026 | 1289 | 1,7E‐04 | 1,7E‐04 | 3,0E‐05 | 3,8E+00 | 3,8E+00 | 4,5E+00 |
| 20139 | Arafura Sea | Central Indo‐Pacific | 1228 | 74 | 4 | 22 | 22,0 | 0,015 | 451 | 1,8E‐04 | 1,8E‐04 | 9,6E‐06 | 3,8E+00 | 3,8E+00 | 5,0E+00 |
| 20232 | Auckland Island | Southern Ocean | 939 | 47 | 2 | 21 | 21,0 | 0,075 | 123 | 6,4E‐03 | 6,4E‐03 | 2,7E‐04 | 2,2E+00 | 2,2E+00 | 3,6E+00 |
| 20029 | Azores Canaries Madeira | Temperate Northern Atlantic | 2642 | 107 | 40 | 14 | 14,3 | 0,005 | 705 | 2,7E‐06 | 2,7E‐06 | 1,0E‐06 | 5,6E+00 | 5,6E+00 | 6,0E+00 |
| 25007 | Baffin Bay ‐ Davis Strait | Arctic | 413 | 38 | 1 | 8 | 8,0 | 0,020 | 86 | 1,9E‐03 | 1,9E‐03 | 5,0E‐05 | 2,7E+00 | 2,7E+00 | 4,3E+00 |
| 20063 | Bahamian | Tropical Atlantic | 3092 | 156 | 13 | 104 | 105,3 | 0,076 | 1288 | 4,8E‐04 | 4,7E‐04 | 4,0E‐05 | 3,3E+00 | 3,3E+00 | 4,4E+00 |
| 20024 | Baltic Sea | Temperate Northern  Atlantic | 1998 | 116 | 92 | 28 | 28,6 | 0,067 | 169 | 1,2E‐04 | 1,2E‐04 | 9,8E‐05 | 3,9E+00 | 3,9E+00 | 4,0E+00 |
| 20131 | Banda Sea | Central Indo‐Pacific | 3291 | 58 | 13 | 212 | 213,3 | 0,062 | 1055 | 9,6E‐04 | 9,5E‐04 | 2,1E‐04 | 3,0E+00 | 3,0E+00 | 3,7E+00 |
| 20205 | Bassian | Temperate Australasia | 6990 | 112 | 78 | 90 | 91,0 | 0,091 | 888 | 1,2E‐04 | 1,2E‐04 | 8,3E‐05 | 3,9E+00 | 3,9E+00 | 4,1E+00 |
| 20011 | Beaufort‐Amundsen‐ Viscount Melville‐Queen Maud | Arctic | 345 | 36 | 1 | 7 | 7,2 | 0,049 | 44 | 7,9E‐03 | 7,7E‐03 | 2,2E‐04 | 2,1E+00 | 2,1E+00 | 3,7E+00 |

| **ECO**  **CODE** | **ECOREGION** | **REALM** | **total**  **species** | **aliens** | **alien**  **(sight)** | **threatened**  **(excl. DD)** | **threatened**  **(incl. DD)** | **Φ_inv** | **assessed** | **EF (main)** | **EF (excl.**  **DD)** | **EF aliens**  **(total)** | **‐log10(EF)** | **‐log10(EF)**  **(excl. DD)** | **‐log10(EF)**  **aliens (total)** |
| --- | --- | --- | --- | --- | --- | --- | --- | --- | --- | --- | --- | --- | --- | --- | --- |
| 20012 | Beaufort Sea ‐  continental coast and shelf | Arctic | 838 | 94 | 6 | 9 | 11,4 | 0,038 | 60 | 1,2E‐03 | 9,5E‐04 | 7,7E‐05 | 2,9E+00 | 3,0E+00 | 4,1E+00 |
| 20062 | Bermuda | Tropical Atlantic | 1472 | 85 | 7 | 36 | 42,0 | 0,038 | 661 | 3,5E‐04 | 3,0E‐04 | 2,9E‐05 | 3,5E+00 | 3,5E+00 | 4,5E+00 |
| 20134 | Bismarck Sea | Central Indo‐Pacific | 4277 | 39 | 6 | 152 | 158,9 | 0,061 | 1393 | 1,2E‐03 | 1,1E‐03 | 1,8E‐04 | 2,9E+00 | 3,0E+00 | 3,7E+00 |
| 20044 | Black Sea | Temperate Northern Atlantic | 1888 | 140 | 84 | 10 | 20,4 | 0,074 | 104 | 1,7E‐04 | 8,4E‐05 | 1,0E‐04 | 3,8E+00 | 4,1E+00 | 4,0E+00 |
| 20141 | Bonaparte Coast | Central Indo‐Pacific | 4768 | 92 | 19 | 271 | 279,1 | 0,046 | 1305 | 5,2E‐04 | 5,0E‐04 | 1,1E‐04 | 3,3E+00 | 3,3E+00 | 4,0E+00 |
| 25230 | Bounty and Antipodes Islands | Southern Ocean | 904 | 45 | 0 | 19 | 20,6 | 0,102 | 110 |  |  | 4,3E‐04 |  |  | 3,4E+00 |
| 20217 | Bouvet Island | Southern Ocean | 303 | 35 | 0 | 8 | 14,7 | 0,164 | 22 |  |  | 3,1E‐03 |  |  | 2,5E+00 |
| 20231 | Campbell Island | Southern Ocean | 796 | 45 | 1 | 21 | 23,9 | 0,069 | 126 | 1,3E‐02 | 1,2E‐02 | 2,9E‐04 | 1,9E+00 | 1,9E+00 | 3,5E+00 |
| 20204 | Cape Howe | Temperate Australasia | 6404 | 92 | 63 | 80 | 85,7 | 0,073 | 1070 | 9,2E‐05 | 8,6E‐05 | 6,3E‐05 | 4,0E+00 | 4,1E+00 | 4,2E+00 |
| 20079 | Cape Verde | Tropical Atlantic | 1296 | 51 | 1 | 10 | 16,1 | 0,039 | 407 | 1,6E‐03 | 9,7E‐04 | 3,0E‐05 | 2,8E+00 | 3,0E+00 | 4,5E+00 |
| 20097 | Cargados Carajos/Tromelin Island | Western Indo‐Pacific | 873 | 42 | 1 | 111 | 124,3 | 0,056 | 567 | 1,2E‐02 | 1,1E‐02 | 2,9E‐04 | 1,9E+00 | 2,0E+00 | 3,5E+00 |
| 25042 | Carolinian | Temperate Northern Atlantic | 4594 | 157 | 63 | 61 | 61,2 | 0,104 | 1285 | 7,9E‐05 | 7,8E‐05 | 3,2E‐05 | 4,1E+00 | 4,1E+00 | 4,5E+00 |
| 20026 | Celtic Seas | Temperate Northern  Atlantic | 6842 | 175 | 120 | 33 | 33,7 | 0,022 | 572 | 1,1E‐05 | 1,1E‐05 | 7,6E‐06 | 5,0E+00 | 5,0E+00 | 5,1E+00 |
| 20177 | Central Chile | Temperate South America | 517 | 20 | 5 | 3 | 4,8 | 0,017 | 104 | 1,6E‐04 | 9,8E‐05 | 3,9E‐05 | 3,8E+00 | 4,0E+00 | 4,4E+00 |
| 20051 | Central Kuroshio Current | Temperate Northern  Pacific | 5640 | 73 | 38 | 119 | 123,2 | 0,024 | 1517 | 5,2E‐05 | 5,0E‐05 | 2,7E‐05 | 4,3E+00 | 4,3E+00 | 4,6E+00 |
| 25199 | Central New Zealand | Temperate Australasia | 4547 | 61 | 40 | 69 | 71,4 | 0,102 | 541 | 3,4E‐04 | 3,2E‐04 | 2,2E‐04 | 3,5E+00 | 3,5E+00 | 3,7E+00 |
| 20175 | Central Peru | Temperate South  America | 1302 | 8 | 3 | 3 | 5,1 | 0,099 | 253 | 6,7E‐04 | 3,9E‐04 | 2,5E‐04 | 3,2E+00 | 3,4E+00 | 3,6E+00 |
| 20093 | Central Somali Coast | Western Indo‐Pacific | 649 | 16 | 3 | 1 | 15,4 | 0,000 | 251 | 0,0E+00 | 0,0E+00 | 0,0E+00 | 0,0E+00 | 0,0E+00 | 0,0E+00 |
| 20106 | Chagos | Western Indo‐Pacific | 1107 | 64 | 3 | 171 | 180,3 | 0,063 | 698 | 5,4E‐03 | 5,1E‐03 | 2,5E‐04 | 2,3E+00 | 2,3E+00 | 3,6E+00 |
| 20187 | Channels and Fjords of Southern Chile | Temperate South America | 1619 | 56 | 10 | 20 | 21,7 | 0,109 | 115 | 2,1E‐03 | 1,9E‐03 | 3,7E‐04 | 2,7E+00 | 2,7E+00 | 3,4E+00 |

| **ECO**  **CODE** | **ECOREGION** | **REALM** | **total**  **species** | **aliens** | **alien**  **(sight)** | **threatened**  **(excl. DD)** | **threatened**  **(incl. DD)** | **Φ_inv** | **assessed** | **EF (main)** | **EF (excl.**  **DD)** | **EF aliens**  **(total)** | **‐log10(EF)** | **‐log10(EF)**  **(excl. DD)** | **‐log10(EF)**  **aliens (total)** |
| --- | --- | --- | --- | --- | --- | --- | --- | --- | --- | --- | --- | --- | --- | --- | --- |
| 25198 | Chatham Island | Temperate Australasia | 1701 | 45 | 0 | 33 | 34,3 | 0,117 | 269 |  |  | 3,3E‐04 |  |  | 3,5E+00 |
| 20167 | Chiapas‐Nicaragua | Tropical Eastern Pacific | 1392 | 64 | 7 | 11 | 21,8 | 0,006 | 669 | 2,7E‐05 | 1,4E‐05 | 3,0E‐06 | 4,6E+00 | 4,9E+00 | 5,5E+00 |
| 20188 | Chiloense | Temperate South  America | 527 | 21 | 4 | 8 | 11,0 | 0,125 | 45 | 7,6E‐03 | 5,5E‐03 | 1,4E‐03 | 2,1E+00 | 2,3E+00 | 2,8E+00 |
| 20013 | Chukchi Sea | Arctic | 732 | 94 | 2 | 18 | 25,8 | 0,058 | 79 | 9,5E‐03 | 6,6E‐03 | 2,0E‐04 | 2,0E+00 | 2,2E+00 | 3,7E+00 |
| 20165 | Clipperton | Tropical Eastern Pacific | 215 | 58 | 2 |  |  | 0,000 | 150 |  |  |  |  |  |  |
| 20120 | Cocos‐Keeling/Christmas Island | Central Indo‐Pacific | 1338 | 56 | 5 | 124 | 128,6 | 0,075 | 651 | 3,0E‐03 | 2,9E‐03 | 2,7E‐04 | 2,5E+00 | 2,5E+00 | 3,6E+00 |
| 20169 | Cocos Islands | Tropical Eastern Pacific | 478 | 49 | 2 | 2 | 9,9 | 0,072 | 349 | 1,0E‐03 | 2,1E‐04 | 4,2E‐05 | 3,0E+00 | 3,7E+00 | 4,4E+00 |
| 20150 | Coral Sea | Central Indo‐Pacific | 2694 | 91 | 10 | 155 | 166,1 | 0,056 | 993 | 9,4E‐04 | 8,8E‐04 | 1,0E‐04 | 3,0E+00 | 3,1E+00 | 4,0E+00 |
| 20060 | Cortezian | Temperate Northern  Pacific | 2268 | 70 | 18 | 16 | 24,4 | 0,004 | 1003 | 6,1E‐06 | 4,0E‐06 | 1,6E‐06 | 5,2E+00 | 5,4E+00 | 5,8E+00 |
| 20215 | Crozet Islands | Southern Ocean | 329 | 41 | 0 | 22 | 29,0 | 0,101 | 64 |  |  | 1,1E‐03 |  |  | 2,9E+00 |
| 20102 | Delagoa | Western Indo‐Pacific | 2997 | 56 | 14 | 77 | 86,8 | 0,025 | 1176 | 1,3E‐04 | 1,2E‐04 | 3,3E‐05 | 3,9E+00 | 3,9E+00 | 4,5E+00 |
| 20124 | East Caroline Islands | Central Indo‐Pacific | 1584 | 37 | 4 | 119 | 129,7 | 0,064 | 833 | 2,5E‐03 | 2,3E‐03 | 2,7E‐04 | 2,6E+00 | 2,6E+00 | 3,6E+00 |
| 20225 | East Antarctic Enderby  Land | Southern Ocean | 471 | 1 | 1 | 14 | 19,5 | 0,144 | 41 | 6,9E‐02 | 4,9E‐02 | 6,9E‐02 | 1,2E+00 | 1,3E+00 | 1,2E+00 |
| 20224 | East Antarctic Wilkes Land | Southern Ocean | 2218 |  | 0 |  |  | 0,164 | 109 |  |  |  |  |  |  |
| 20226 | East Antarctic Dronning  Maud Land | Southern Ocean | 460 |  | 0 |  |  | 0,135 | 27 |  |  |  |  |  |  |
| 20052 | East China Sea | Temperate Northern Pacific | 3715 | 92 | 44 | 55 | 57,5 | 0,009 | 1304 | 9,2E‐06 | 8,8E‐06 | 4,4E‐06 | 5,0E+00 | 5,1E+00 | 5,4E+00 |
| 20003 | East Greenland Shelf | Arctic | 767 | 62 | 2 | 12 | 16,5 | 0,042 | 111 | 3,2E‐03 | 2,3E‐03 | 1,0E‐04 | 2,5E+00 | 2,6E+00 | 4,0E+00 |
| 20015 | East Siberian Sea | Arctic | 445 | 14 | 0 | 1 | 12,1 | 0,050 | 8 |  |  | 5,4E‐03 |  |  | 2,3E+00 |
| 20163 | Easter Island | Eastern Indo‐Pacific | 220 | 15 | 0 | 6 | 9,0 | 0,138 | 65 |  |  | 1,3E‐03 |  |  | 2,9E+00 |
| 25014 | Eastern Bering Sea | Arctic | 1309 | 87 | 6 | 21 | 23,9 | 0,064 | 194 | 1,3E‐03 | 1,2E‐03 | 9,1E‐05 | 2,9E+00 | 2,9E+00 | 4,0E+00 |
| 20076 | Eastern Brazil | Tropical Atlantic | 3467 | 77 | 30 | 31 | 32,9 | 0,038 | 686 | 6,1E‐05 | 5,7E‐05 | 2,4E‐05 | 4,2E+00 | 4,2E+00 | 4,6E+00 |
| 20064 | Eastern Caribbean | Tropical Atlantic | 3372 | 146 | 22 | 120 | 122,7 | 0,094 | 1170 | 4,5E‐04 | 4,4E‐04 | 6,7E‐05 | 3,3E+00 | 3,4E+00 | 4,2E+00 |

| **ECO**  **CODE** | **ECOREGION** | **REALM** | **total**  **species** | **aliens** | **alien**  **(sight)** | **threatened**  **(excl. DD)** | **threatened**  **(incl. DD)** | **Φ_inv** | **assessed** | **EF (main)** | **EF (excl.**  **DD)** | **EF aliens**  **(total)** | **‐log10(EF)** | **‐log10(EF)**  **(excl. DD)** | **‐log10(EF)**  **aliens (total)** |
| --- | --- | --- | --- | --- | --- | --- | --- | --- | --- | --- | --- | --- | --- | --- | --- |
| 20173 | Eastern Galapagos Islands | Tropical Eastern Pacific | 1085 | 46 | 2 | 13 | 18,6 | 0,044 | 516 | 8,0E‐04 | 5,6E‐04 | 3,5E‐05 | 3,1E+00 | 3,3E+00 | 4,5E+00 |
| 20107 | Eastern India | Western Indo‐Pacific | 2709 | 20 | 12 | 20 | 23,3 | 0,052 | 602 | 1,7E‐04 | 1,4E‐04 | 1,0E‐04 | 3,8E+00 | 3,8E+00 | 4,0E+00 |
| 20127 | Eastern Philippines | Central Indo‐Pacific | 6747 | 36 | 12 | 155 | 166,6 | 0,047 | 2232 | 2,9E‐04 | 2,7E‐04 | 9,7E‐05 | 3,5E+00 | 3,6E+00 | 4,0E+00 |
| 20144 | Exmouth to Broome | Central Indo‐Pacific | 7215 | 91 | 21 | 323 | 327,3 | 0,049 | 2291 | 3,4E‐04 | 3,3E‐04 | 7,7E‐05 | 3,5E+00 | 3,5E+00 | 4,1E+00 |
| 20021 | Faroe Plateau | Temperate Northern  Atlantic | 799 | 100 | 15 | 9 | 14,5 | 0,027 | 64 | 4,1E‐04 | 2,6E‐04 | 6,2E‐05 | 3,4E+00 | 3,6E+00 | 4,2E+00 |
| 20074 | Fernando de Naronha and Atoll das Rocas | Tropical Atlantic | 529 | 60 | 6 | 11 | 14,1 | 0,047 | 187 | 5,9E‐04 | 4,6E‐04 | 5,9E‐05 | 3,2E+00 | 3,3E+00 | 4,2E+00 |
| 25147 | Fiji Islands | Central Indo‐Pacific | 3599 | 43 | 8 | 144 | 145,9 | 0,048 | 1251 | 7,1E‐04 | 7,0E‐04 | 1,3E‐04 | 3,2E+00 | 3,2E+00 | 3,9E+00 |
| 20070 | Floridian | Tropical Atlantic | 9472 | 181 | 102 | 134 | 138,3 | 0,073 | 1693 | 5,9E‐05 | 5,7E‐05 | 3,3E‐05 | 4,2E+00 | 4,2E+00 | 4,5E+00 |
| 25154 | Gilbert/Ellis Islands | Eastern Indo‐Pacific | 1369 | 64 | 1 | 57 | 59,5 | 0,054 | 649 | 4,9E‐03 | 4,7E‐03 | 7,7E‐05 | 2,3E+00 | 2,3E+00 | 4,1E+00 |
| 20208 | Great Australian Bight | Temperate Australasia | 1871 | 73 | 11 | 27 | 29,5 | 0,036 | 508 | 1,9E‐04 | 1,7E‐04 | 2,9E‐05 | 3,7E+00 | 3,8E+00 | 4,5E+00 |
| 20171 | Guayaquil | Tropical Eastern Pacific | 1397 | 12 | 4 | 7 | 20,8 | 0,009 | 561 | 8,1E‐05 | 2,7E‐05 | 2,7E‐05 | 4,1E+00 | 4,6E+00 | 4,6E+00 |
| 20071 | Guianan | Tropical Atlantic | 1960 | 104 | 12 | 23 | 31,4 | 0,014 | 832 | 4,4E‐05 | 3,2E‐05 | 5,0E‐06 | 4,4E+00 | 4,5E+00 | 5,3E+00 |
| 20089 | Gulf of Aden | Western Indo‐Pacific | 2022 | 11 | 4 | 187 | 191,8 | 0,059 | 668 | 4,2E‐03 | 4,1E‐03 | 1,5E‐03 | 2,4E+00 | 2,4E+00 | 2,8E+00 |
| 20054 | Gulf of Alaska | Temperate Northern Pacific | 1591 | 112 | 22 | 18 | 25,7 | 0,106 | 170 | 7,2E‐04 | 5,1E‐04 | 1,4E‐04 | 3,1E+00 | 3,3E+00 | 3,8E+00 |
| 20083 | Gulf of Guinea Central | Tropical Atlantic | 1149 | 28 | 4 | 6 | 8,7 | 0,004 | 596 | 1,3E‐05 | 9,0E‐06 | 1,9E‐06 | 4,9E+00 | 5,0E+00 | 5,7E+00 |
| 20084 | Gulf of Guinea Islands | Tropical Atlantic | 576 | 64 | 2 | 5 | 26,0 | 0,009 | 340 | 3,5E‐04 | 6,6E‐05 | 1,1E‐05 | 3,5E+00 | 4,2E+00 | 5,0E+00 |
| 20082 | Gulf of Guinea Upwelling | Tropical Atlantic | 978 | 58 | 4 | 8 | 11,9 | 0,004 | 502 | 2,6E‐05 | 1,7E‐05 | 1,8E‐06 | 4,6E+00 | 4,8E+00 | 5,8E+00 |
| 25081 | Gulf of Guinea West | Tropical Atlantic | 1057 | 61 | 7 | 4 | 12,5 | 0,004 | 559 | 1,3E‐05 | 4,2E‐06 | 1,5E‐06 | 4,9E+00 | 5,4E+00 | 5,8E+00 |
| 25040 | Gulf of Maine/Bay of  Fundy | Temperate Northern  Atlantic | 3800 | 157 | 64 | 34 | 37,9 | 0,064 | 772 | 4,9E‐05 | 4,4E‐05 | 2,0E‐05 | 4,3E+00 | 4,4E+00 | 4,7E+00 |
| 20091 | Gulf of Oman | Western Indo‐Pacific | 953 | 14 | 4 | 34 | 44,4 | 0,011 | 287 | 4,2E‐04 | 3,2E‐04 | 1,2E‐04 | 3,4E+00 | 3,5E+00 | 3,9E+00 |
| 20138 | Gulf of Papua | Central Indo‐Pacific | 856 | 77 | 4 | 57 | 64,5 | 0,019 | 519 | 6,0E‐04 | 5,3E‐04 | 3,1E‐05 | 3,2E+00 | 3,3E+00 | 4,5E+00 |

| **ECO**  **CODE** | **ECOREGION** | **REALM** | **total**  **species** | **aliens** | **alien**  **(sight)** | **threatened**  **(excl. DD)** | **threatened**  **(incl. DD)** | **Φ_inv** | **assessed** | **EF (main)** | **EF (excl.**  **DD)** | **EF aliens**  **(total)** | **‐log10(EF)** | **‐log10(EF)**  **(excl. DD)** | **‐log10(EF)**  **aliens (total)** |
| --- | --- | --- | --- | --- | --- | --- | --- | --- | --- | --- | --- | --- | --- | --- | --- |
| 20037 | Gulf of St. Lawrence ‐  Eastern Scotian Shelf | Temperate Northern  Atlantic | 2379 | 98 | 26 | 21 | 24,6 | 0,079 | 394 | 1,9E‐04 | 1,6E‐04 | 5,0E‐05 | 3,7E+00 | 3,8E+00 | 4,3E+00 |
| 20115 | Gulf of Thailand | Central Indo‐Pacific | 1267 | 27 | 7 | 126 | 131,1 | 0,020 | 719 | 5,2E‐04 | 5,0E‐04 | 1,3E‐04 | 3,3E+00 | 3,3E+00 | 3,9E+00 |
| 20112 | Gulf of Tonkin | Central Indo‐Pacific | 1511 | 45 | 17 | 6 | 8,4 | 0,004 | 367 | 5,0E‐06 | 3,6E‐06 | 1,9E‐06 | 5,3E+00 | 5,4E+00 | 5,7E+00 |
| 20129 | Halmahera | Central Indo‐Pacific | 1148 | 38 | 3 | 107 | 114,2 | 0,065 | 404 | 6,1E‐03 | 5,7E‐03 | 4,8E‐04 | 2,2E+00 | 2,2E+00 | 3,3E+00 |
| 25152 | Hawaii | Eastern Indo‐Pacific | 3947 | 164 | 101 | 89 | 93,4 | 0,065 | 1145 | 5,3E‐05 | 5,0E‐05 | 3,2E‐05 | 4,3E+00 | 4,3E+00 | 4,5E+00 |
| 20213 | Heard and Macdonald Islands | Southern Ocean | 475 | 68 | 0 | 27 | 27,4 | 0,121 | 94 |  |  | 5,2E‐04 |  |  | 3,3E+00 |
| 20211 | Houtman | Temperate Australasia | 2773 | 83 | 31 | 178 | 180,9 | 0,047 | 756 | 3,6E‐04 | 3,6E‐04 | 1,4E‐04 | 3,4E+00 | 3,4E+00 | 3,9E+00 |
| 20008 | Hudson Complex | Arctic | 945 | 27 | 2 | 12 | 23,8 | 0,045 | 80 | 6,7E‐03 | 3,4E‐03 | 5,0E‐04 | 2,2E+00 | 2,5E+00 | 3,3E+00 |
| 20176 | Humboldtian | Temperate South  America | 1196 | 20 | 6 | 5 | 7,3 | 0,030 | 184 | 2,0E‐04 | 1,4E‐04 | 6,0E‐05 | 3,7E+00 | 3,9E+00 | 4,2E+00 |
| 20179 | Juan Fernandez and Desventuradas | Temperate South America | 385 | 20 | 1 | 4 | 5,6 | 0,017 | 140 | 6,8E‐04 | 4,8E‐04 | 3,4E‐05 | 3,2E+00 | 3,3E+00 | 4,5E+00 |
| 25046 | Kamchatka Shelf and  Coast | Temperate Northern  Pacific | 606 | 103 | 2 | 5 | 8,1 | 0,022 | 81 | 1,1E‐03 | 6,9E‐04 | 2,2E‐05 | 3,0E+00 | 3,2E+00 | 4,7E+00 |
| 20017 | Kara Sea | Arctic | 648 | 14 | 0 | 1 | 3,2 | 0,000 | 11 |  |  | 0,0E+00 |  |  | 0,0E+00 |
| 20214 | Kerguelen Islands | Southern Ocean | 830 | 68 | 0 | 28 | 32,2 | 0,055 | 143 |  |  | 1,8E‐04 |  |  | 3,7E+00 |
| 25195 | Kermadec Island | Temperate Australasia | 1205 | 46 | 2 | 41 | 42,8 | 0,103 | 187 | 1,2E‐02 | 1,1E‐02 | 5,1E‐04 | 1,9E+00 | 1,9E+00 | 3,3E+00 |
| 25009 | Lancaster Sound | Arctic | 226 | 27 | 0 | 7 | 8,4 | 0,042 | 46 |  |  | 2,8E‐04 |  |  | 3,5E+00 |
| 20016 | Laptev Sea | Arctic | 547 | 14 | 0 | 7 | 20,9 | 0,059 | 34 |  |  | 2,6E‐03 |  |  | 2,6E+00 |
| 20209 | Leeuwin | Temperate Australasia | 4075 | 91 | 33 | 75 | 76,0 | 0,036 | 747 | 1,1E‐04 | 1,1E‐04 | 4,0E‐05 | 4,0E+00 | 4,0E+00 | 4,4E+00 |
| 20132 | Lesser Sunda | Central Indo‐Pacific | 2558 | 81 | 11 | 216 | 217,9 | 0,047 | 1229 | 7,5E‐04 | 7,4E‐04 | 1,0E‐04 | 3,1E+00 | 3,1E+00 | 4,0E+00 |
| 20155 | Line Islands | Eastern Indo‐Pacific | 1383 | 117 | 10 | 128 | 131,6 | 0,065 | 709 | 1,2E‐03 | 1,2E‐03 | 1,0E‐04 | 2,9E+00 | 2,9E+00 | 4,0E+00 |
| 20151 | Lord Howe and Norfolk Islands | Central Indo‐Pacific | 2872 | 107 | 6 | 144 | 147,5 | 0,062 | 905 | 1,7E‐03 | 1,6E‐03 | 9,4E‐05 | 2,8E+00 | 2,8E+00 | 4,0E+00 |
| 20212 | Macquarie Island | Southern Ocean | 763 | 49 | 0 | 32 | 37,7 | 0,140 | 139 |  |  | 7,7E‐04 |  |  | 3,1E+00 |
| 20061 | Magdalena Transition | Temperate Northern Pacific | 838 | 66 | 13 | 4 | 20,1 | 0,000 | 526 | 0,0E+00 | 0,0E+00 | 0,0E+00 | 0,0E+00 | 0,0E+00 | 0,0E+00 |
| 20118 | Malacca Strait | Central Indo‐Pacific | 1352 | 32 | 9 | 82 | 86,6 | 0,032 | 519 | 6,0E‐04 | 5,6E‐04 | 1,7E‐04 | 3,2E+00 | 3,2E+00 | 3,8E+00 |

| **ECO**  **CODE** | **ECOREGION** | **REALM** | **total**  **species** | **aliens** | **alien**  **(sight)** | **threatened**  **(excl. DD)** | **threatened**  **(incl. DD)** | **Φ_inv** | **assessed** | **EF (main)** | **EF (excl.**  **DD)** | **EF aliens**  **(total)** | **‐log10(EF)** | **‐log10(EF)**  **(excl. DD)** | **‐log10(EF)**  **aliens (total)** |
| --- | --- | --- | --- | --- | --- | --- | --- | --- | --- | --- | --- | --- | --- | --- | --- |
| 20105 | Maldives | Western Indo‐Pacific | 3328 | 70 | 9 | 193 | 195,5 | 0,048 | 992 | 1,1E‐03 | 1,0E‐03 | 1,4E‐04 | 3,0E+00 | 3,0E+00 | 3,9E+00 |
| 20186 | Malvinas/Falklands | Temperate South America | 1403 | 81 | 7 | 22 | 23,8 | 0,109 | 89 | 4,2E‐03 | 3,9E‐03 | 3,6E‐04 | 2,4E+00 | 2,4E+00 | 3,4E+00 |
| 20203 | Manning‐Hawkesbury | Temperate Australasia | 7405 | 96 | 60 | 88 | 89,2 | 0,075 | 1370 | 8,1E‐05 | 8,0E‐05 | 5,1E‐05 | 4,1E+00 | 4,1E+00 | 4,3E+00 |
| 20123 | Mariana Islands | Central Indo‐Pacific | 1995 | 117 | 13 | 127 | 132,3 | 0,068 | 840 | 8,2E‐04 | 7,9E‐04 | 9,1E‐05 | 3,1E+00 | 3,1E+00 | 4,0E+00 |
| 20158 | Tuamotus | Eastern Indo‐Pacific | 1364 | 89 | 2 | 28 | 28,2 | 0,040 | 646 | 8,7E‐04 | 8,6E‐04 | 2,0E‐05 | 3,1E+00 | 3,1E+00 | 4,7E+00 |
| 20153 | Marshall Islands | Eastern Indo‐Pacific | 1806 | 105 | 7 | 186 | 189,6 | 0,065 | 868 | 2,0E‐03 | 2,0E‐03 | 1,3E‐04 | 2,7E+00 | 2,7E+00 | 3,9E+00 |
| 20098 | Mascarene Islands | Western Indo‐Pacific | 3000 | 44 | 8 | 217 | 218,6 | 0,075 | 1238 | 1,7E‐03 | 1,6E‐03 | 3,0E‐04 | 2,8E+00 | 2,8E+00 | 3,5E+00 |
| 20166 | Mexican Tropical Pacific | Tropical Eastern Pacific | 1075 | 64 | 9 | 9 | 14,2 | 0,001 | 607 | 2,0E‐06 | 1,3E‐06 | 2,8E‐07 | 5,7E+00 | 5,9E+00 | 6,6E+00 |
| 20191 | Namaqua | Temperate Southern  Africa | 2454 | 36 | 12 | 20 | 21,0 | 0,027 | 623 | 7,6E‐05 | 7,2E‐05 | 2,5E‐05 | 4,1E+00 | 4,1E+00 | 4,6E+00 |
| 25190 | Namib | Temperate Southern Africa | 1282 | 24 | 8 | 14 | 16,1 | 0,010 | 395 | 5,1E‐05 | 4,4E‐05 | 1,7E‐05 | 4,3E+00 | 4,4E+00 | 4,8E+00 |
| 20193 | Natal | Temperate Southern  Africa | 4188 | 33 | 14 | 50 | 51,4 | 0,036 | 1235 | 1,1E‐04 | 1,0E‐04 | 4,5E‐05 | 4,0E+00 | 4,0E+00 | 4,3E+00 |
| 20149 | New Caledonia | Central Indo‐Pacific | 8956 | 99 | 15 | 173 | 178,1 | 0,046 | 1693 | 3,2E‐04 | 3,1E‐04 | 4,9E‐05 | 3,5E+00 | 3,5E+00 | 4,3E+00 |
| 20168 | Nicoya | Tropical Eastern Pacific | 1404 | 49 | 9 | 11 | 21,1 | 0,000 | 819 | 0,0E+00 | 0,0E+00 | 0,0E+00 | 0,0E+00 | 0,0E+00 | 0,0E+00 |
| 20145 | Ningaloo | Central Indo‐Pacific | 2769 | 83 | 13 | 182 | 188,8 | 0,049 | 876 | 8,1E‐04 | 7,8E‐04 | 1,3E‐04 | 3,1E+00 | 3,1E+00 | 3,9E+00 |
| 25055 | North American Pacific  Fijordland | Temperate Northern  Pacific | 2531 | 133 | 47 | 19 | 29,1 | 0,038 | 232 | 1,0E‐04 | 6,6E‐05 | 3,6E‐05 | 4,0E+00 | 4,2E+00 | 4,4E+00 |
| 20001 | North Greenland | Arctic | 495 | 41 | 0 | 8 | 21,6 | 0,030 | 30 |  |  | 5,2E‐04 |  |  | 3,3E+00 |
| 25184 | North Patagonian Gulfs | Temperate South  America | 582 | 60 | 21 | 7 | 10,6 | 0,156 | 109 | 7,2E‐04 | 4,8E‐04 | 2,5E‐04 | 3,1E+00 | 3,3E+00 | 3,6E+00 |
| 20133 | Northeast Sulawesi | Central Indo‐Pacific | 319 | 8 | 5 | 76 | 78,2 | 0,070 | 128 | 8,6E‐03 | 8,3E‐03 | 5,4E‐03 | 2,1E+00 | 2,1E+00 | 2,3E+00 |
| 20075 | Northeastern Brazil | Tropical Atlantic | 1947 | 101 | 21 | 22 | 28,1 | 0,040 | 453 | 1,2E‐04 | 9,2E‐05 | 2,5E‐05 | 3,9E+00 | 4,0E+00 | 4,6E+00 |
| 20048 | Northeastern Honshu | Temperate Northern Pacific | 1617 | 57 | 10 | 3 | 12,9 | 0,001 | 314 | 4,2E‐06 | 9,7E‐07 | 7,3E‐07 | 5,4E+00 | 6,0E+00 | 6,1E+00 |
| 25196 | Northeastern New  Zealand | Temperate Australasia | 4043 | 59 | 42 | 49 | 55,0 | 0,102 | 440 | 3,0E‐04 | 2,7E‐04 | 2,2E‐04 | 3,5E+00 | 3,6E+00 | 3,7E+00 |

| **ECO**  **CODE** | **ECOREGION** | **REALM** | **total**  **species** | **aliens** | **alien**  **(sight)** | **threatened**  **(excl. DD)** | **threatened**  **(incl. DD)** | **Φ_inv** | **assessed** | **EF (main)** | **EF (excl.**  **DD)** | **EF aliens**  **(total)** | **‐log10(EF)** | **‐log10(EF)**  **(excl. DD)** | **‐log10(EF)**  **aliens (total)** |
| --- | --- | --- | --- | --- | --- | --- | --- | --- | --- | --- | --- | --- | --- | --- | --- |
| 20087 | Northern and Central Red  Sea | Western Indo‐Pacific | 1971 | 73 | 21 | 206 | 211,6 | 0,061 | 808 | 7,6E‐04 | 7,4E‐04 | 2,2E‐04 | 3,1E+00 | 3,1E+00 | 3,7E+00 |
| 20108 | Northern Bay of Bengal | Western Indo‐Pacific | 1149 | 17 | 7 | 11 | 14,6 | 0,014 | 333 | 8,5E‐05 | 6,4E‐05 | 3,5E‐05 | 4,1E+00 | 4,2E+00 | 4,5E+00 |
| 25058 | Northern California | Temperate Northern  Pacific | 3212 | 200 | 148 | 46 | 48,7 | 0,073 | 491 | 4,9E‐05 | 4,6E‐05 | 3,6E‐05 | 4,3E+00 | 4,3E+00 | 4,4E+00 |
| 20172 | Northern Galapagos Islands | Tropical Eastern Pacific | 225 | 46 | 1 | 2 | 3,4 | 0,059 | 159 | 1,3E‐03 | 7,4E‐04 | 2,7E‐05 | 2,9E+00 | 3,1E+00 | 4,6E+00 |
| 20005 | Northern Grand Banks ‐  Southern Labrador | Arctic | 667 | 71 | 7 | 15 | 18,6 | 0,031 | 180 | 4,6E‐04 | 3,7E‐04 | 4,6E‐05 | 3,3E+00 | 3,4E+00 | 4,3E+00 |
| 20043 | Northern Gulf of Mexico | Temperate Northern Atlantic | 9189 | 143 | 88 | 110 | 112,3 | 0,043 | 1736 | 3,1E‐05 | 3,1E‐05 | 1,9E‐05 | 4,5E+00 | 4,5E+00 | 4,7E+00 |
| 20006 | Northern Labrador | Arctic | 1038 | 28 | 2 | 12 | 12,9 | 0,028 | 132 | 1,4E‐03 | 1,3E‐03 | 9,9E‐05 | 2,9E+00 | 2,9E+00 | 4,0E+00 |
| 20122 | Ogasawara Islands | Central Indo‐Pacific | 897 | 55 | 1 | 27 | 28,6 | 0,047 | 327 | 4,1E‐03 | 3,9E‐03 | 7,5E‐05 | 2,4E+00 | 2,4E+00 | 4,1E+00 |
| 25057 | Oregon, Washington, Vancouver Coast and  Shelf | Temperate Northern Pacific | 2397 | 182 | 108 | 28 | 29,5 | 0,030 | 350 | 2,3E‐05 | 2,2E‐05 | 1,4E‐05 | 4,6E+00 | 4,7E+00 | 4,9E+00 |
| 20047 | Oyashio Current | Temperate Northern Pacific | 1232 | 57 | 3 | 3 | 4,7 | 0,006 | 122 | 7,5E‐05 | 4,8E‐05 | 3,9E‐06 | 4,1E+00 | 4,3E+00 | 5,4E+00 |
| 20126 | Palawan/North Borneo | Central Indo‐Pacific | 3583 | 33 | 5 | 132 | 132,4 | 0,028 | 1459 | 5,1E‐04 | 5,1E‐04 | 7,8E‐05 | 3,3E+00 | 3,3E+00 | 4,1E+00 |
| 20170 | Panama Bight | Tropical Eastern Pacific | 2575 | 58 | 17 | 29 | 30,0 | 0,015 | 1071 | 2,4E‐05 | 2,3E‐05 | 7,1E‐06 | 4,6E+00 | 4,6E+00 | 5,1E+00 |
| 20130 | Papua | Central Indo‐Pacific | 1428 | 44 | 4 | 150 | 150,0 | 0,066 | 672 | 3,7E‐03 | 3,7E‐03 | 3,3E‐04 | 2,4E+00 | 2,4E+00 | 3,5E+00 |
| 25185 | Patagonian Shelf | Temperate South America | 1482 | 88 | 13 | 19 | 23,5 | 0,092 | 104 | 1,6E‐03 | 1,3E‐03 | 2,4E‐04 | 2,8E+00 | 2,9E+00 | 3,6E+00 |
| 20218 | Peter the First Island | Southern Ocean | 120 |  | 0 |  |  | 0,298 | 20 |  |  |  |  |  |  |
| 20156 | Phoenix/Tokelau/Norther n Cook Islands | Eastern Indo‐Pacific | 1202 | 121 | 7 | 98 | 102,1 | 0,062 | 581 | 1,6E‐03 | 1,5E‐03 | 9,1E‐05 | 2,8E+00 | 2,8E+00 | 4,0E+00 |
| 20216 | Prince Edward Islands | Southern Ocean | 448 | 26 | 0 | 18 | 21,5 | 0,172 | 43 |  |  | 3,3E‐03 |  |  | 2,5E+00 |
| 25056 | Puget Trough/Georgia Basin | Temperate Northern Pacific | 1876 | 133 | 82 | 18 | 29,1 | 0,039 | 181 | 7,7E‐05 | 4,7E‐05 | 4,7E‐05 | 4,1E+00 | 4,3E+00 | 4,3E+00 |
| 20159 | Rapa‐Pitcairn | Eastern Indo‐Pacific | 603 | 70 | 0 | 83 | 85,5 | 0,067 | 346 |  |  | 2,4E‐04 |  |  | 3,6E+00 |

| **ECO**  **CODE** | **ECOREGION** | **REALM** | **total**  **species** | **aliens** | **alien**  **(sight)** | **threatened**  **(excl. DD)** | **threatened**  **(incl. DD)** | **Φ_inv** | **assessed** | **EF (main)** | **EF (excl.**  **DD)** | **EF aliens**  **(total)** | **‐log10(EF)** | **‐log10(EF)**  **(excl. DD)** | **‐log10(EF)**  **aliens (total)** |
| --- | --- | --- | --- | --- | --- | --- | --- | --- | --- | --- | --- | --- | --- | --- | --- |
| 20164 | Revillagigedos | Tropical Eastern Pacific | 403 | 61 | 1 | 2 | 3,7 | 0,000 | 328 | 0,0E+00 | 0,0E+00 | 0,0E+00 | 0,0E+00 | 0,0E+00 | 0,0E+00 |
| 20182 | Rio de la Plata | Temperate South America | 363 | 46 | 20 | 4 | 11,7 | 0,245 | 59 | 2,4E‐03 | 8,3E‐04 | 1,1E‐03 | 2,6E+00 | 3,1E+00 | 3,0E+00 |
| 20181 | Rio Grande | Temperate South  America | 842 | 64 | 14 | 9 | 11,2 | 0,083 | 274 | 2,4E‐04 | 1,9E‐04 | 5,3E‐05 | 3,6E+00 | 3,7E+00 | 4,3E+00 |
| 20229 | Ross Sea | Southern Ocean | 1761 |  | 0 |  |  | 0,051 | 46 |  |  |  |  |  |  |
| 20028 | Saharan Upwelling | Temperate Northern  Atlantic | 1873 | 83 | 10 | 9 | 11,2 | 0,017 | 569 | 3,3E‐05 | 2,7E‐05 | 4,0E‐06 | 4,5E+00 | 4,6E+00 | 5,4E+00 |
| 20157 | Samoa Islands | Eastern Indo‐Pacific | 2058 | 109 | 11 | 160 | 161,3 | 0,091 | 907 | 1,5E‐03 | 1,5E‐03 | 1,5E‐04 | 2,8E+00 | 2,8E+00 | 3,8E+00 |
| 20073 | Sao Pedro and Sao Paulo  Islands | Tropical Atlantic | 382 | 92 | 2 | 2 | 5,0 | 0,222 | 149 | 3,8E‐03 | 1,5E‐03 | 8,2E‐05 | 2,4E+00 | 2,8E+00 | 4,1E+00 |
| 20039 | Scotian Shelf | Temperate Northern Atlantic | 2181 | 78 | 27 | 21 | 23,4 | 0,029 | 656 | 3,8E‐05 | 3,4E‐05 | 1,3E‐05 | 4,4E+00 | 4,5E+00 | 4,9E+00 |
| 20049 | Sea of Japan/East Sea | Temperate Northern  Pacific | 1921 | 45 | 17 | 8 | 11,8 | 0,006 | 224 | 1,8E‐05 | 1,2E‐05 | 6,7E‐06 | 4,8E+00 | 4,9E+00 | 5,2E+00 |
| 20045 | Sea of Okhotsk | Temperate Northern Pacific | 606 | 39 | 3 | 7 | 8,7 | 0,022 | 71 | 9,2E‐04 | 7,4E‐04 | 7,1E‐05 | 3,0E+00 | 3,1E+00 | 4,1E+00 |
| 20096 | Seychelles | Western Indo‐Pacific | 2867 | 20 | 8 | 269 | 269,0 | 0,062 | 1226 | 1,7E‐03 | 1,7E‐03 | 6,8E‐04 | 2,8E+00 | 2,8E+00 | 3,2E+00 |
| 20210 | Shark Bay | Temperate Australasia | 2072 | 79 | 9 | 105 | 105,0 | 0,040 | 799 | 5,9E‐04 | 5,9E‐04 | 6,7E‐05 | 3,2E+00 | 3,2E+00 | 4,2E+00 |
| 20201 | Snares Island | Temperate Australasia | 783 | 46 | 1 | 20 | 21,3 | 0,114 | 127 | 1,9E‐02 | 1,8E‐02 | 4,2E‐04 | 1,7E+00 | 1,7E+00 | 3,4E+00 |
| 20161 | Society Islands | Eastern Indo‐Pacific | 1882 | 43 | 7 | 73 | 74,3 | 0,048 | 668 | 7,6E‐04 | 7,4E‐04 | 1,2E‐04 | 3,1E+00 | 3,1E+00 | 3,9E+00 |
| 20135 | Solomon Archipelago | Central Indo‐Pacific | 3236 | 40 | 2 | 84 | 84,6 | 0,060 | 1032 | 2,5E‐03 | 2,5E‐03 | 1,2E‐04 | 2,6E+00 | 2,6E+00 | 3,9E+00 |
| 20136 | Solomon Sea | Central Indo‐Pacific | 1952 | 78 | 3 | 86 | 86,6 | 0,057 | 876 | 1,9E‐03 | 1,9E‐03 | 7,2E‐05 | 2,7E+00 | 2,7E+00 | 4,1E+00 |
| 20207 | South Australian Gulfs | Temperate Australasia | 2929 | 80 | 33 | 55 | 61,8 | 0,037 | 567 | 1,2E‐04 | 1,1E‐04 | 5,0E‐05 | 3,9E+00 | 4,0E+00 | 4,3E+00 |
| 20114 | South China Sea Oceanic Islands | Central Indo‐Pacific | 1475 | 64 | 23 | 75 | 85,0 | 0,036 | 765 | 1,8E‐04 | 1,6E‐04 | 6,3E‐05 | 3,8E+00 | 3,8E+00 | 4,2E+00 |
| 20027 | South European Atlantic  Shelf | Temperate Northern  Atlantic | 5117 | 145 | 87 | 21 | 31,3 | 0,018 | 543 | 1,2E‐05 | 8,2E‐06 | 7,3E‐06 | 4,9E+00 | 5,1E+00 | 5,1E+00 |
| 20220 | South Georgia | Southern Ocean | 1756 | 61 | 1 | 21 | 22,4 | 0,141 | 91 | 3,5E‐02 | 3,2E‐02 | 5,7E‐04 | 1,5E+00 | 1,5E+00 | 3,2E+00 |
| 20104 | South India and Sri Lanka | Western Indo‐Pacific | 4662 | 23 | 12 | 131 | 134,5 | 0,039 | 1103 | 4,0E‐04 | 3,9E‐04 | 2,1E‐04 | 3,4E+00 | 3,4E+00 | 3,7E+00 |

| **ECO**  **CODE** | **ECOREGION** | **REALM** | **total**  **species** | **aliens** | **alien**  **(sight)** | **threatened**  **(excl. DD)** | **threatened**  **(incl. DD)** | **Φ_inv** | **assessed** | **EF (main)** | **EF (excl.**  **DD)** | **EF aliens**  **(total)** | **‐log10(EF)** | **‐log10(EF)**  **(excl. DD)** | **‐log10(EF)**  **aliens (total)** |
| --- | --- | --- | --- | --- | --- | --- | --- | --- | --- | --- | --- | --- | --- | --- | --- |
| 20121 | South Kuroshio | Central Indo‐Pacific | 4438 | 57 | 18 | 221 | 223,8 | 0,042 | 2012 | 2,6E‐04 | 2,5E‐04 | 8,1E‐05 | 3,6E+00 | 3,6E+00 | 4,1E+00 |
| 20221 | South Orkney Islands | Southern Ocean | 1127 | 17 | 0 | 14 | 15,6 | 0,182 | 68 |  |  | 2,5E‐03 |  |  | 2,6E+00 |
| 20219 | South Sandwich Islands | Southern Ocean | 819 | 61 | 1 | 19 | 27,1 | 0,144 | 59 | 6,6E‐02 | 4,6E‐02 | 1,1E‐03 | 1,2E+00 | 1,3E+00 | 3,0E+00 |
| 20222 | South Shetland Islands | Southern Ocean | 2104 | 17 | 0 | 18 | 24,8 | 0,056 | 102 |  |  | 8,1E‐04 |  |  | 3,1E+00 |
| 20099 | Southeast Madagascar | Western Indo‐Pacific | 364 | 41 | 2 | 12 | 18,8 | 0,030 | 140 | 2,0E‐03 | 1,3E‐03 | 9,7E‐05 | 2,7E+00 | 2,9E+00 | 4,0E+00 |
| 20137 | Southeast Papua New Guinea | Central Indo‐Pacific | 1398 | 77 | 10 | 79 | 84,8 | 0,047 | 619 | 6,4E‐04 | 5,9E‐04 | 8,3E‐05 | 3,2E+00 | 3,2E+00 | 4,1E+00 |
| 20180 | Southeastern Brazil | Temperate South  America | 3143 | 95 | 64 | 26 | 29,2 | 0,012 | 592 | 9,3E‐06 | 8,3E‐06 | 6,3E‐06 | 5,0E+00 | 5,1E+00 | 5,2E+00 |
| 25059 | Southern California Bight | Temperate Northern Pacific | 3197 | 185 | 100 | 40 | 43,3 | 0,068 | 716 | 4,1E‐05 | 3,8E‐05 | 2,2E‐05 | 4,4E+00 | 4,4E+00 | 4,7E+00 |
| 20066 | Southern Caribbean | Tropical Atlantic | 5119 | 141 | 60 | 98 | 102,4 | 0,165 | 1262 | 2,2E‐04 | 2,1E‐04 | 9,5E‐05 | 3,7E+00 | 3,7E+00 | 4,0E+00 |
| 20113 | Southern China | Central Indo‐Pacific | 2633 | 53 | 28 | 29 | 36,8 | 0,022 | 969 | 3,0E‐05 | 2,3E‐05 | 1,6E‐05 | 4,5E+00 | 4,6E+00 | 4,8E+00 |
| 20038 | Southern Grand Banks ‐  South Newfoundland | Temperate Northern Atlantic | 675 | 93 | 10 | 14 | 19,5 | 0,049 | 231 | 4,1E‐04 | 3,0E‐04 | 4,4E‐05 | 3,4E+00 | 3,5E+00 | 4,4E+00 |
| 20069 | Southern Gulf of Mexico | Tropical Atlantic | 7483 | 139 | 60 | 107 | 111,9 | 0,068 | 1413 | 9,0E‐05 | 8,6E‐05 | 3,9E‐05 | 4,0E+00 | 4,1E+00 | 4,4E+00 |
| 20200 | South New Zealand | Temperate Australasia | 2210 | 51 | 17 | 48 | 50,2 | 0,092 | 270 | 1,0E‐03 | 9,6E‐04 | 3,3E‐04 | 3,0E+00 | 3,0E+00 | 3,5E+00 |
| 20088 | Southern Red Sea | Western Indo‐Pacific | 1069 | 10 | 5 | 58 | 59,0 | 0,015 | 494 | 3,6E‐04 | 3,6E‐04 | 1,8E‐04 | 3,4E+00 | 3,4E+00 | 3,7E+00 |
| 20116 | Southern Vietnam | Central Indo‐Pacific | 1097 | 24 | 3 | 8 | 9,5 | 0,006 | 611 | 3,1E‐05 | 2,6E‐05 | 3,8E‐06 | 4,5E+00 | 4,6E+00 | 5,4E+00 |
| 20067 | Southwestern Caribbean | Tropical Atlantic | 4492 | 113 | 32 | 118 | 119,6 | 0,058 | 1477 | 1,5E‐04 | 1,4E‐04 | 4,1E‐05 | 3,8E+00 | 3,8E+00 | 4,4E+00 |
| 20078 | St. Helena and Ascension  Islands | Tropical Atlantic | 477 | 61 | 0 | 6 | 6,0 | 0,007 | 164 |  |  | 4,2E‐06 |  |  | 5,4E+00 |
| 20128 | Sulawesi Sea/Makassar Strait | Central Indo‐Pacific | 1895 | 14 | 3 | 204 | 204,0 | 0,064 | 746 | 5,8E‐03 | 5,8E‐03 | 1,2E‐03 | 2,2E+00 | 2,2E+00 | 2,9E+00 |
| 20095 | East African Coral Coast | Western Indo‐Pacific | 3456 | 46 | 17 | 324 | 324,0 | 0,056 | 1378 | 7,7E‐04 | 7,7E‐04 | 2,8E‐04 | 3,1E+00 | 3,1E+00 | 3,5E+00 |
| 20094 | Northern Monsoon Current Coast | Western Indo‐Pacific | 552 | 16 | 4 | 189 | 190,1 | 0,070 | 269 | 1,2E‐02 | 1,2E‐02 | 3,1E‐03 | 1,9E+00 | 1,9E+00 | 2,5E+00 |

| **ECO**  **CODE** | **ECOREGION** | **REALM** | **total**  **species** | **aliens** | **alien**  **(sight)** | **threatened**  **(excl. DD)** | **threatened**  **(incl. DD)** | **Φ_inv** | **assessed** | **EF (main)** | **EF (excl.**  **DD)** | **EF aliens**  **(total)** | **‐log10(EF)** | **‐log10(EF)**  **(excl. DD)** | **‐log10(EF)**  **aliens (total)** |
| --- | --- | --- | --- | --- | --- | --- | --- | --- | --- | --- | --- | --- | --- | --- | --- |
| 20101 | Bight of Sofala/Swamp  Coast | Western Indo‐Pacific | 861 | 37 | 4 | 69 | 70,1 | 0,034 | 446 | 1,3E‐03 | 1,3E‐03 | 1,4E‐04 | 2,9E+00 | 2,9E+00 | 3,8E+00 |
| 20197 | Three Kings‐North Cape | Temperate Australasia | 2151 | 74 | 17 | 29 | 29,0 | 0,144 | 313 | 7,9E‐04 | 7,9E‐04 | 1,8E‐04 | 3,1E+00 | 3,1E+00 | 3,7E+00 |
| 20146 | Tonga Islands | Central Indo‐Pacific | 1794 | 102 | 7 | 41 | 42,9 | 0,024 | 865 | 1,7E‐04 | 1,6E‐04 | 1,1E‐05 | 3,8E+00 | 3,8E+00 | 4,9E+00 |
| 20077 | Trindade and Martin Vaz Islands | Tropical Atlantic | 507 | 36 | 0 | 9 | 10,3 | 0,045 | 169 |  |  | 7,6E‐05 |  |  | 4,1E+00 |
| 20189 | Tristan Gough | Temperate South  America | 326 | 61 | 0 | 11 | 12,1 | 0,184 | 76 |  |  | 4,8E‐04 |  |  | 3,3E+00 |
| 20162 | Marquesas | Eastern Indo‐Pacific | 826 | 38 | 2 | 9 | 9,3 | 0,036 | 369 | 4,5E‐04 | 4,3E‐04 | 2,4E‐05 | 3,3E+00 | 3,4E+00 | 4,6E+00 |
| 20202 | Tweed‐Moreton | Temperate Australasia | 7624 | 92 | 37 | 182 | 182,3 | 0,048 | 1609 | 1,5E‐04 | 1,5E‐04 | 5,9E‐05 | 3,8E+00 | 3,8E+00 | 4,2E+00 |
| 20183 | Uruguay‐Buenos Aires Shelf | Temperate South America | 951 | 89 | 24 | 16 | 16,0 | 0,033 | 164 | 1,3E‐04 | 1,3E‐04 | 3,6E‐05 | 3,9E+00 | 3,9E+00 | 4,4E+00 |
| 20148 | Vanuatu | Central Indo‐Pacific | 3964 | 47 | 5 | 42 | 42,0 | 0,023 | 1326 | 1,5E‐04 | 1,5E‐04 | 1,5E‐05 | 3,8E+00 | 3,8E+00 | 4,8E+00 |
| 20041 | Virginian | Temperate Northern Atlantic | 5125 | 163 | 97 | 52 | 52,6 | 0,055 | 1189 | 2,5E‐05 | 2,5E‐05 | 1,5E‐05 | 4,6E+00 | 4,6E+00 | 4,8E+00 |
| 20125 | West Caroline Islands | Central Indo‐Pacific | 2462 | 41 | 6 | 138 | 139,3 | 0,061 | 1282 | 1,1E‐03 | 1,1E‐03 | 1,6E‐04 | 3,0E+00 | 3,0E+00 | 3,8E+00 |
| 20227 | Weddell Sea | Southern Ocean | 1826 | 1 | 1 | 6 | 7,3 | 0,141 | 46 | 2,2E‐02 | 1,8E‐02 | 2,2E‐02 | 1,6E+00 | 1,7E+00 | 1,6E+00 |
| 20004 | West Greenland Shelf | Arctic | 718 | 74 | 3 | 9 | 9,6 | 0,024 | 85 | 9,0E‐04 | 8,4E‐04 | 3,6E‐05 | 3,0E+00 | 3,1E+00 | 4,4E+00 |
| 20100 | Western and Northern Madagascar | Western Indo‐Pacific | 4810 | 47 | 12 | 376 | 376,3 | 0,068 | 1765 | 1,2E‐03 | 1,2E‐03 | 3,1E‐04 | 2,9E+00 | 2,9E+00 | 3,5E+00 |
| 20092 | Western Arabian Sea | Western Indo‐Pacific | 819 | 8 | 4 | 22 | 22,0 | 0,046 | 187 | 1,3E‐03 | 1,3E‐03 | 6,7E‐04 | 2,9E+00 | 2,9E+00 | 3,2E+00 |
| 20206 | Western Bassian | Temperate Australasia | 2821 | 87 | 39 | 71 | 72,2 | 0,077 | 562 | 2,5E‐04 | 2,5E‐04 | 1,1E‐04 | 3,6E+00 | 3,6E+00 | 3,9E+00 |
| 20068 | Western Caribbean | Tropical Atlantic | 1932 | 97 | 15 | 94 | 95,1 | 0,088 | 835 | 6,7E‐04 | 6,6E‐04 | 1,0E‐04 | 3,2E+00 | 3,2E+00 | 4,0E+00 |
| 20174 | Western Galapagos Islands | Tropical Eastern Pacific | 435 | 43 | 2 | 5 | 7,7 | 0,023 | 319 | 2,8E‐04 | 1,8E‐04 | 1,3E‐05 | 3,6E+00 | 3,7E+00 | 4,9E+00 |
| 20103 | Western India | Western Indo‐Pacific | 3514 | 26 | 18 | 57 | 59,1 | 0,028 | 688 | 1,4E‐04 | 1,3E‐04 | 9,4E‐05 | 3,9E+00 | 3,9E+00 | 4,0E+00 |
| 20050 | Yellow Sea | Temperate Northern Pacific | 1712 | 76 | 39 | 6 | 8,5 | 0,001 | 142 | 1,6E‐06 | 1,2E‐06 | 8,5E‐07 | 5,8E+00 | 5,9E+00 | 6,1E+00 |

| **ECO**  **CODE** | **ECOREGION** | **REALM** | **total**  **species** | **aliens** | **alien**  **(sight)** | **threatened**  **(excl. DD)** | **threatened**  **(incl. DD)** | **Φ_inv** | **assessed** | **EF (main)** | **EF (excl.**  **DD)** | **EF aliens**  **(total)** | **‐log10(EF)** | **‐log10(EF)**  **(excl. DD)** | **‐log10(EF)**  **aliens (total)** |
| --- | --- | --- | --- | --- | --- | --- | --- | --- | --- | --- | --- | --- | --- | --- | --- |
| 20119 | Southern Java | Central Indo‐Pacific | 1047 | 55 | 4 | 51 | 55,8 | 0,023 | 513 | 6,3E‐04 | 5,8E‐04 | 4,6E‐05 | 3,2E+00 | 3,2E+00 | 4,3E+00 |
| 20111 | Western Sumatra | Western Indo‐Pacific | 753 | 21 | 4 | 43 | 45,9 | 0,056 | 357 | 1,8E‐03 | 1,7E‐03 | 3,4E‐04 | 2,7E+00 | 2,8E+00 | 3,5E+00 |
| 20142 | Torres Strait Northern Great Barrier Reef | Central Indo‐Pacific | 7000 | 83 | 20 | 357 | 369,0 | 0,056 | 2010 | 5,1E‐04 | 5,0E‐04 | 1,2E‐04 | 3,3E+00 | 3,3E+00 | 3,9E+00 |
| 20143 | Central and Southern Great Barrier Reef | Central Indo‐Pacific | 9827 | 89 | 35 | 388 | 392,4 | 0,058 | 2175 | 3,0E‐04 | 3,0E‐04 | 1,2E‐04 | 3,5E+00 | 3,5E+00 | 3,9E+00 |
| 20117 | Sunda Shelf/Java Sea | Central Indo‐Pacific | 1692 | 29 | 9 | 141 | 142,1 | 0,040 | 820 | 7,6E‐04 | 7,6E‐04 | 2,4E‐04 | 3,1E+00 | 3,1E+00 | 3,6E+00 |
| 20002 | North and East Iceland | Arctic | 543 | 64 | 3 | 5 | 6,6 | 0,027 | 41 | 1,4E‐03 | 1,1E‐03 | 6,8E‐05 | 2,8E+00 | 3,0E+00 | 4,2E+00 |
| 20020 | South and West Iceland | Temperate Northern  Atlantic | 1089 | 81 | 7 | 12 | 13,4 | 0,037 | 113 | 6,2E‐04 | 5,6E‐04 | 5,4E‐05 | 3,2E+00 | 3,3E+00 | 4,3E+00 |
| 20025 | North Sea | Temperate Northern Atlantic | 7916 | 252 | 206 | 51 | 53,1 | 0,025 | 440 | 1,5E‐05 | 1,4E‐05 | 1,2E‐05 | 4,8E+00 | 4,9E+00 | 4,9E+00 |
| 20022 | Southern Norway | Temperate Northern  Atlantic | 2190 | 108 | 24 | 23 | 25,3 | 0,039 | 150 | 2,7E‐04 | 2,5E‐04 | 6,0E‐05 | 3,6E+00 | 3,6E+00 | 4,2E+00 |
| 20023 | Northern Norway and Finnmark | Temperate Northern Atlantic | 2221 | 28 | 4 | 19 | 24,3 | 0,032 | 133 | 1,5E‐03 | 1,2E‐03 | 2,1E‐04 | 2,8E+00 | 2,9E+00 | 3,7E+00 |
| 20018 | North and East Barents  Sea | Arctic | 1956 | 31 | 2 | 6 | 11,5 | 0,033 | 61 | 3,1E‐03 | 1,6E‐03 | 2,0E‐04 | 2,5E+00 | 2,8E+00 | 3,7E+00 |
| 20019 | White Sea | Arctic | 832 | 8 | 2 | 5 | 10,6 | 0,030 | 16 | 9,9E‐03 | 4,7E‐03 | 2,5E‐03 | 2,0E+00 | 2,3E+00 | 2,6E+00 |
| 20065 | Greater Antilles | Tropical Atlantic | 5843 | 184 | 52 | 128 | 133,5 | 0,065 | 1602 | 1,0E‐04 | 1,0E‐04 | 2,9E‐05 | 4,0E+00 | 4,0E+00 | 4,5E+00 |
| 20160 | Southern Cook/Austral Islands | Eastern Indo‐Pacific | 993 | 105 | 5 | 42 | 46,7 | 0,068 | 504 | 1,3E‐03 | 1,1E‐03 | 6,0E‐05 | 2,9E+00 | 2,9E+00 | 4,2E+00 |
| 25085 | Gulf of Guinea South | Tropical Atlantic | 1170 | 27 | 5 | 7 | 11,9 | 0,002 | 640 | 9,0E‐06 | 5,3E‐06 | 1,7E‐06 | 5,0E+00 | 5,3E+00 | 5,8E+00 |
| 25086 | Angolan | Tropical Atlantic | 1259 | 20 | 5 | 6 | 18,8 | 0,008 | 616 | 5,0E‐05 | 1,6E‐05 | 1,2E‐05 | 4,3E+00 | 4,8E+00 | 4,9E+00 |
| 25080 | Sahelian Upwelling | Tropical Atlantic | 1136 | 55 | 7 | 7 | 8,0 | 0,005 | 579 | 9,4E‐06 | 8,2E‐06 | 1,2E‐06 | 5,0E+00 | 5,1E+00 | 5,9E+00 |
| 20030 | Adriatic Sea | Temperate Northern Atlantic | 2695 | 129 | 83 | 16 | 19,2 | 0,114 | 247 | 1,1E‐04 | 8,9E‐05 | 6,8E‐05 | 4,0E+00 | 4,1E+00 | 4,2E+00 |
| 25032 | Levantine Sea | Temperate Northern  Atlantic | 2541 | 215 | 168 | 11 | 13,6 | 0,018 | 329 | 4,4E‐06 | 3,6E‐06 | 3,4E‐06 | 5,4E+00 | 5,4E+00 | 5,5E+00 |
| 25033 | Tunisian Plateau/Gulf of Sidra | Temperate Northern Atlantic | 961 | 119 | 37 | 8 | 13,0 | 0,016 | 308 | 1,9E‐05 | 1,1E‐05 | 5,8E‐06 | 4,7E+00 | 4,9E+00 | 5,2E+00 |
| 25034 | Ionian Sea | Temperate Northern  Atlantic | 2429 | 163 | 64 | 9 | 11,0 | 0,017 | 267 | 1,1E‐05 | 9,1E‐06 | 4,4E‐06 | 5,0E+00 | 5,0E+00 | 5,4E+00 |

| **ECO**  **CODE** | **ECOREGION** | **REALM** | **total**  **species** | **aliens** | **alien**  **(sight)** | **threatened**  **(excl. DD)** | **threatened**  **(incl. DD)** | **Φ_inv** | **assessed** | **EF (main)** | **EF (excl.**  **DD)** | **EF aliens**  **(total)** | **‐log10(EF)** | **‐log10(EF)**  **(excl. DD)** | **‐log10(EF)**  **aliens (total)** |
| --- | --- | --- | --- | --- | --- | --- | --- | --- | --- | --- | --- | --- | --- | --- | --- |
| 25031 | Aegean Sea | Temperate Northern  Atlantic | 3050 | 175 | 118 | 13 | 17,7 | 0,013 | 235 | 8,1E‐06 | 6,0E‐06 | 5,5E‐06 | 5,1E+00 | 5,2E+00 | 5,3E+00 |
| 25036 | Alboran Sea | Temperate Northern Atlantic | 1322 | 109 | 35 | 18 | 22,0 | 0,027 | 328 | 5,2E‐05 | 4,2E‐05 | 1,7E‐05 | 4,3E+00 | 4,4E+00 | 4,8E+00 |
| 25035 | Western Mediterranean | Temperate Northern  Atlantic | 5651 | 221 | 162 | 27 | 32,9 | 0,025 | 636 | 8,1E‐06 | 6,6E‐06 | 5,9E‐06 | 5,1E+00 | 5,2E+00 | 5,2E+00 |
| 25010 | High Arctic Archipelago | Arctic | 207 | 43 | 0 | 3 | 4,7 | 0,024 | 17 |  |  | 1,5E‐04 |  |  | 3,8E+00 |

# References for Supporting Information

Borgelt, J., Dorber, M., Høiberg, M. A., & Verones, F. (2022). More than half of data deficient species predicted to be threatened by extinction. *Communications Biology*, *5*(1), 679. https://doi.org/10.1038/s42003-022-03638-9

Veron, J. E. N., Stafford-Smith, M. G., Turak, E., & DeVantier, L. M. (2016). *Corals of the World*. http://www.coralsoftheworld.org/page/home/
